# Multi-omics analyses revealed key factors involved in fluorescent carbon-dots-regulated secondary metabolism in *Tetrastigma hemsleyanum*

**DOI:** 10.1186/s12951-022-01271-6

**Published:** 2022-02-02

**Authors:** Xin Peng, Zhuomi Xie, Xiuhua Wang, Yuxiang Zhao, Chuyun Yang, Zhongyi Zhang, Mingjie Li, Jianping Zheng, Yuhui Wang

**Affiliations:** 1 Ningbo Research Institute of Zhejiang University, Ningbo, 315100 People’s Republic of China; 2 Fujian Agriculture and Forestry University, Fuzhou, 350028 People’s Republic of China; 3 Cixi Institute of Biomedical Engineering, Ningbo Institute of Materials Technology and Engineering, Chinese Academy of Sciences, Ningbo, 315300 People’s Republic of China

**Keywords:** Fluorescent carbon-dots, *Tetrastigma hemsleyanum*, Quality ingredients, Hub genes

## Abstract

**Background:**

Luminescent nanomaterials (LNMs), especially newly-exploited fluorescent carbon-dots (CDs), have demonstrated promising candidates for sunlight harvesting and enhanced photosynthesis efficiency of crops. However, most of the studies focus on the design and synthesis of LNMs and primary metabolism in biomass acceleration, secondary metabolism that closely associated with the quality ingredients of plants is rarely mentioned.

**Results:**

UV-absorptive and water-soluble NIR-CDs were harvested via a facile microwave-assisted carbonization method. The effect and regulatory mechanism of NIR-CDs on the secondary metabolism and bioactive ingredients accumulation in *Tetrastigma hemsleyanum* were explored. A total of 191 differential secondary metabolites and 6874 differentially expressed genes were identified when the NIR-CDs were adopted for enhancing growth of *T. hemsleyanum*. The phenolic acids were generally improved, but the flavonoids were more likely to decrease. The pivotal differentially expressed genes were involved in biosynthesis of secondary metabolites, flavonoid biosynthesis, porphyrin and chlorophyll metabolism, etc. The gene-metabolite association was constructed and 44 hub genes highly related to quality ingredients accumulation and growth were identified, among which and the top 5 genes of the PPI network might be the key regulators.

**Conclusion:**

This research provided key regulatory genes and differentially accumulating quality ingredients under NIR-CDs-treatment, which could provide a theoretical basis for expanding the applications of nanomaterial in industrial crop agriculture.

**Graphical Abstract:**

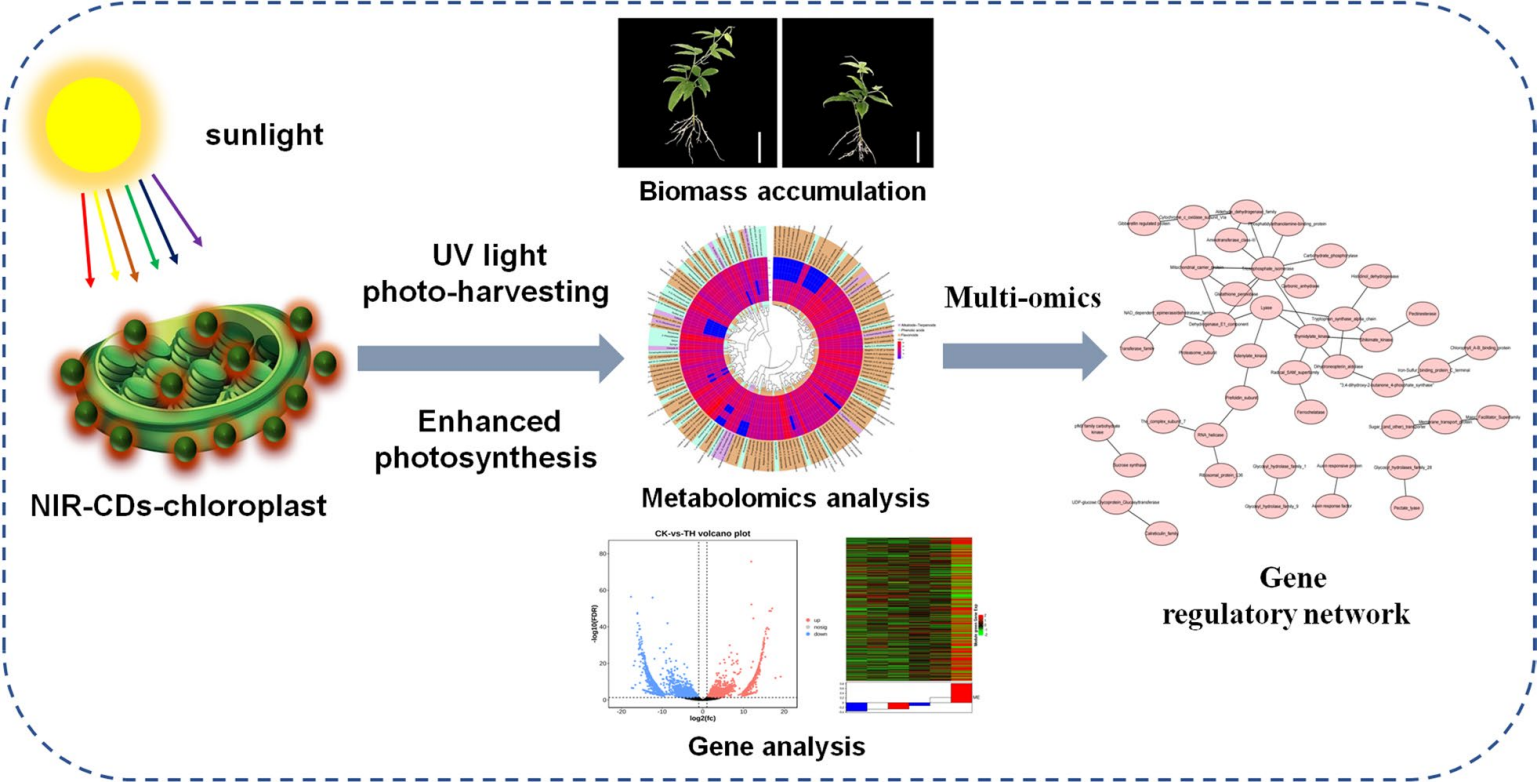

**Supplementary Information:**

The online version contains supplementary material available at 10.1186/s12951-022-01271-6.

## Background

At present, the whole world has reached a consensus that an effective and sustainable agricultural system is essential to the existence and development of humanity. Aiming to sustainably meet future demands for food, new agri-tech revolutions including information technology, new materials, and methodologies have largely emerged [1–4]. Among them, engineered nanomaterials (ENMs)-based nanotechnology positively leads this variation, which enhances the efficiency of cropping systems associated with all inputs via more efficient delivery mechanisms, and thereby improves the efficiency of nutrient utilization, and better management of disease and crop loss [5].

Photosynthesis is the most important energy conversion process on the earth due to its massive and green utilization of sunlight. ENMs reveals great potential to elevate plant photosynthesis efficiency through the following strategies i.e. avoiding abiotic stress [6], increasing chlorophyll content [7], enhancing sunlight harvesting and utilization [8], scavenging reactive oxygen species [9] and promoting electron transfer [10]. In virtue of photoluminescence character, luminescent nanomaterials (LNMs) are ideal candidates for the sunlight absorption and photon use [11]. Because of their small size and environmentally responsive release property, these LNMs possess the characteristics of good penetrability and diffusion in the plant vasculature after root or foliar applications [12]. Many types of LNMs, such as representative semiconductor-type quantum dots (QDs), fluorescent carbon dots (CDs), metal nanoclusters, lanthanide-doped up-conversion phosphors (UCPs), have been demonstrated great potentials in enhancing photon absorption in ultraviolet, red or near-infrared (NIR) region of the sunlight and elevated photosynthesis efficiency and crop yield [11]. Positive progresses have been achieved especially on the design or hybridization of these LNMs. For example, toxicity-reduced MPA-capped CdSe QDs, Si-QDs, PEI-coated UCPs, UCPs@CDs nanocomposites and diverse CDs (blue CDs-chloroplasts hybrid, bule/red dual-emissive CDs and NIR or far-red CDs) have been gradually developed and constructed for the enhanced sunlight absorption and photosynthesis efficiency [13–21]. Among the forementioned LNMs, as a newly-emerged zero-dimension nanophosphors, fluorescent CDs have attracted more attentions due to their fascinating merits e.g. facile and low-cost preparation, low toxicity, good physicochemical stability, excellent optical properties [22]. In our recently-reported work [23], we have produced the first comprehensive analysis of the light-harvesting effects and related mechanisms of NIR-CDs (as a preferential-selected LNMs) on primary metabolism of plant at gene level, which provides a method to research the mechanism of LNMs-caused increased sunlight absorption and photosynthesis efficiency. However, previous researches mainly focused on the design and fabrication of light-absorption nanomaterials, and the effect of these LNMs on growth of plant. There often exists an antagonistic effect between primary and secondary metabolism in plants, so more rapid photosynthesis may lead to the lower accumulation of nutritional or bioactive ingredients in plants. ENMs may be an effective approach to ensure vigorous primary metabolism that could remain a high level of biomass growth rate, but it is also essential and urgent to monitor the responses of secondary metabolism that would significantly influence the quality ingredients in plants [5, 11].

In recent years, technical advancements in metabolomics integrated with transcriptomics analyses have provided effective ways to explore the complex regulatory network in plants [24]. Weighted gene co-expression network analysis (WGCNA), a recently appeared approach in systems biology has been gradually applied to describe gene-related patterns under different treatment conditions, and to explore the key candidate genes involved in multiple metabolic pathways. Particularly, it has become an important tool in the identification of hub genes and the gene-metabolite association, then rebuild the gene regulatory sub-networks.

Herein, as a continued research work, the effect of NIR-CDs on the secondary metabolism and bioactive ingredients of *Tetrastigma hemsleyanum* (*T. hemsleyanum*, a class of medicinal plant) was determined, and the possible mechanism was also further explored by an integrated transcriptome and metabolome research. The gene-metabolite association were constructed by WGCNA, then the candidate hub genes highly related to quality ingredients accumulation and growth were identified. The results would lay the foundation for the application of nanotechnology in industrial crop agriculture.

## Results

### Characterizations of the NIR-CDs

**Fig. 1:**
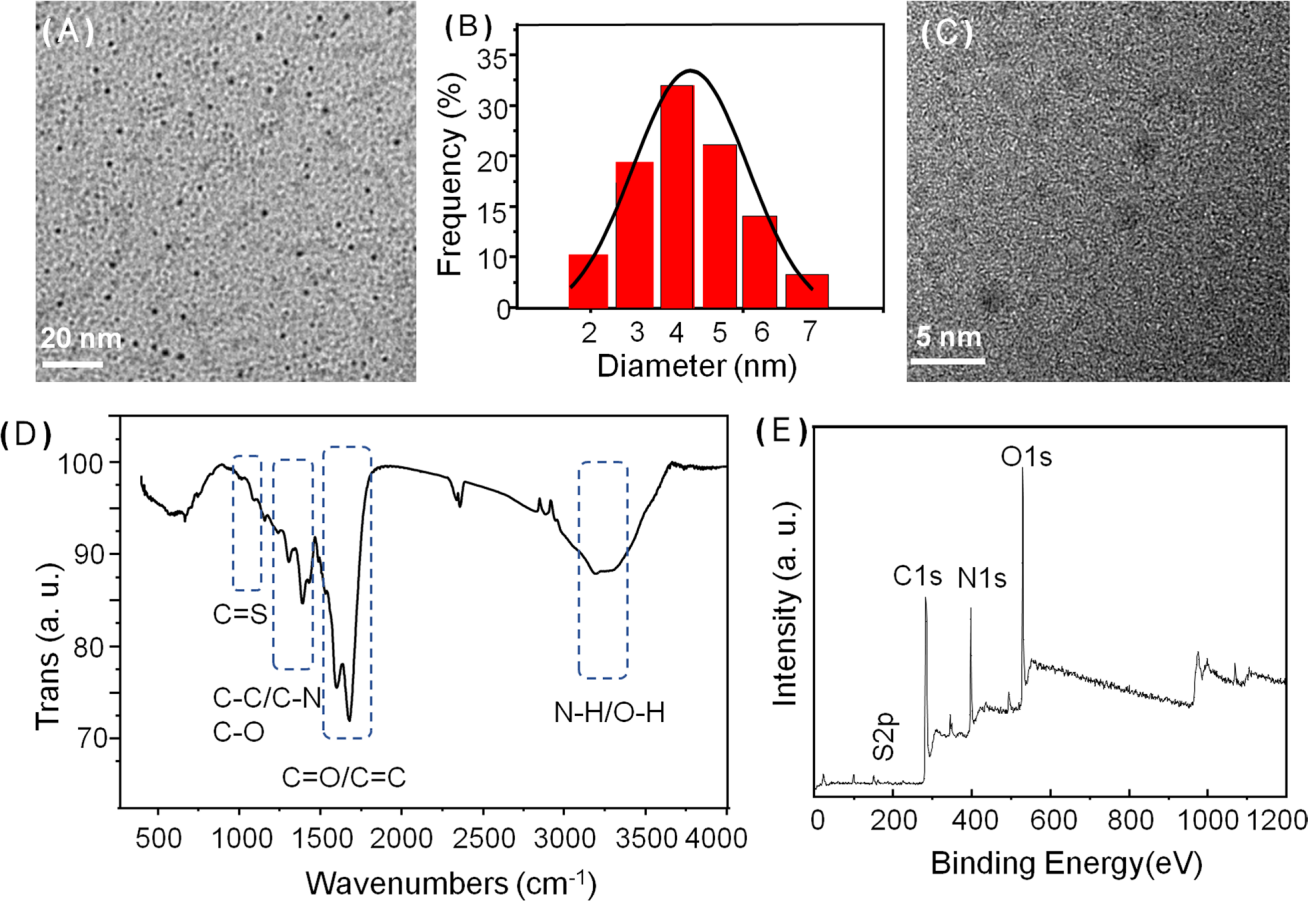
The TEM image (**A**), particle distribution (**B**), HR-TEM image (**C**), FT-IR spectrum (**D**) and XPS testing (**E**) of the harvested NIR-CDs

NIR-CDs were obtained through a microwave-assisted carbonization manner following our previous work using GSH and formamide as the starting materials [25]. Firstly, the structure and morphology of the harvested NIR-CDs were characterized by a series of measurements of TEM, XRD, Raman spectrum. As seen from the TEM image (Fig. 1A), the NIR-CDs present very good mono-dispersity, uniform spherical morphologies, and a narrow particle distribution with a mean size of 3.9 nm (Fig. 1B). Unfortunately, the NIR-CDs are mostly noncrystalline, which is clearly confirmed by the no obvious lattice fringes of HR-TEM image (Fig. 1C). In the XRD pattern (Additional file 1: Fig. S1a), a typical peak at 26° is assigned to the (002) plane of graphite, which further verifies the noncrystalline graphite structure of the NIR-CDs [26]. Besides, the graphite structure of NIR-CDs is also confirmed by the Raman spectrum (Additional file 1: Fig. S1b). As shown therein, two obvious peaks centered at 1550 and 1342 cm^−1^ i.e. G-band and D-band are attributed to sp^2^ and sp^3^ hybridization of carbon-atom. Simultaneously, a low ratio of D to G further demonstrates the dominate pristine carbon in the NIR-CDs [27].

Next, frequently-used FT-IR spectrum, XPS and Zeta potential measurements were performed to investigate surface state of the NIR-CDs. As shown in Fig. 1D, a typical and strong absorption band from 3100 to 3500 cm^−1^ is observed and attributed to the stretching vibration of O–H and N–H. The representative stretching vibrations absorption peaks of C=O and C=C/C=N are assigned to 1689 and 1583 cm^−1^, respectively. The peaks centered at 1160, 1249 and 1390 cm^−1^ are the stretching vibration absorptions of C–C, C–O and C–N, respectively. The absorption band (1000–1100 cm^−1^) is assigned to C=S and oxidized S bonds [28]. These FT-IR assignments are further clearly confirmed by XPS analysis (Fig. 1E). Representative peaks centered at 283, 398, 529, 161 eV are assigned to C 1 s, N 1 s, O 1 s and S 2p, indicating that the NIR-CDs mainly comprise of C, N, O and S elements (atom ratio, C:O:N:S = 63.9:16.3:19.4:0.4). In high resolution spectrum of C 1 s (Additional file 2: Fig. S2a), four typical peaks at 284.8, 286.3, 288.0 and 290.1 are found, which is attributed to C=C/C−C, C−N/C−O, C=N/C=O and N−C=O, respectively [29]. High resolution N 1 s spectrum is fitted to be three peaks of pyridine-like N, graphitic N, and pyrrole-like N that severally centered at 398.5, 400.0 and 402.6 eV (Additional file 2: Fig. S2b) [30]. In O 1 s spectrum, the two peaks at 531.3 and 533.6 eV belong to C−OH and C=O, [31], respectively (Additional file 2: Fig. S2c). Besides, in S 2p spectrum, four binding energies at 162.2, 163.4, 164.8 and 169.7 are identified, corresponding to thiolate, 2p_3/2_ and 2p_1/2_ of thiophene S, and oxidized S, respectively [32]. Furthermore, zeta potential measurement illustrates that the NIR-CDs are negatively charged (ζ =  − 19.2 mV, Additional file 3: Fig. S3), which is ascribed to the large number of surface hydroxyl and amino groups. The abundance of hydrophilic groups will enable strong electrostatic exclusion and good colloid stability of the NIR-CDs.

**Fig. 2:**
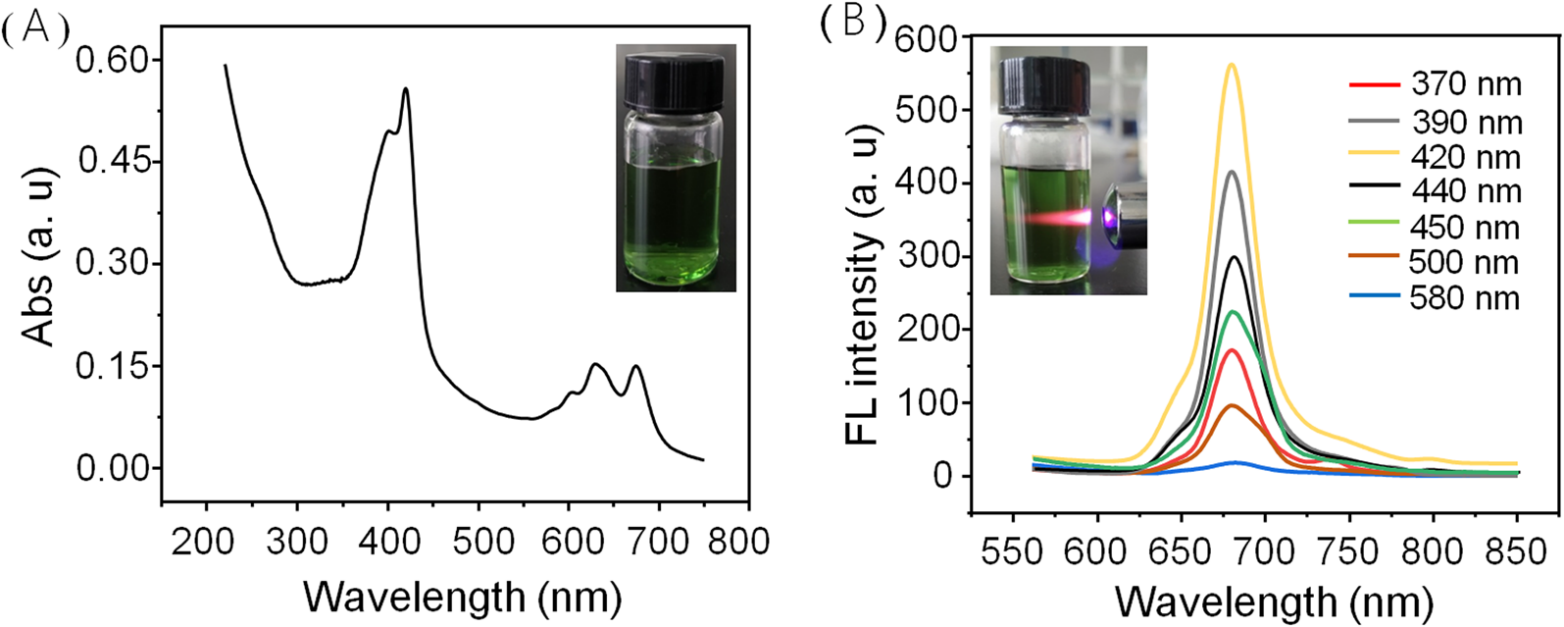
**A** Uv–vis absorption of the NIR-CDs (100 µg/mL), inset: digital photograph of the NIR-CDs solution under daylight. **B** Fluorescence emission spectra of the NIR-CDs (20 µg/mL) under different excitation wavelengths, inset: digital photograph of the NIR-CDs solution under an excitation of laser pointer (405 nm)

Subsequently, the optical properties including Uv–vis absorption, fluorescence spectrum and fluorescence lifetime of the NIR-CDs were inspected, respectively. As shown in Fig. 2A, three obvious absorption bands (240–300 nm, 350–450 nm, and 550–750 nm) are observed, which are usually classified to the typical π → π* transition of the aromatic C=C bond, π → π* and n → π* transitions of the aromatic π system including C=O, C=N, and C=S bonds [28, 33, 34], respectively. In Fig. 2b, the CDs show brightly NIR emission range from 620 to 750 nm with a narrow peak centered at 680 nm under varying excitation wavelengths. Such an excitation-independent fluorescence emission property is mostly attributed to the surface states/defects induced emission [35]. In addition, the mean fluorescence lifetime of the NIR-CDs is measured and quantified to be 3.2 ns with bi-exponential decays (Additional file 4: Fig. S4), and the absolute fluorescence quantum yield is measured to be 18.4% under the optimal excitation of 420 nm. In our previous reports [23, 25], the UV-absorptive and NIR emissive CDs have been demonstrated excellent tolerance to photobleaching, good storage stability at ambient environment and competitive agent in light-harvesting and electron transfer from photosystem II (PS II) to photosystem I (PS I) in chloroplasts [18, 36]. Therefore, NIR-CDs is an ideal light-conversion phosphor model for the research of LNMs-mediated photosynthesis promotion, secondary metabolism of crop and the corresponding possible mechanism.

As a potential nanomaterial used in agriculture, the toxicity or safety of NIR-CDs should be estimated. Finally, the cytotoxicity measurement of NIR-CDs was performed using a classical MTT method. As shown in (see in Additional file 5: Fig. S5), after being incubated with diverse concentrations of NIR-CDs (0, 25, 50, 75, 100, 150 and 200 µg/mL), the viabilities of two-selected cell lines i.e. human bronchial epithelial cells (16HBE) and MCF-7 were higher than 90%, implying that the NIR-CDs revealed low toxicity to mammalian cells(Additional file 5: Fig. S5). Furthermore, the recent advances for in vivo bioimaging and theranostics have also demonstrated the excellent biosafety of these NIR-CDs [37–39].

### NIR-CDs treatment significantly improved growth and enhanced photosynthesis of T. hemsleyanum

**Fig. 3:**
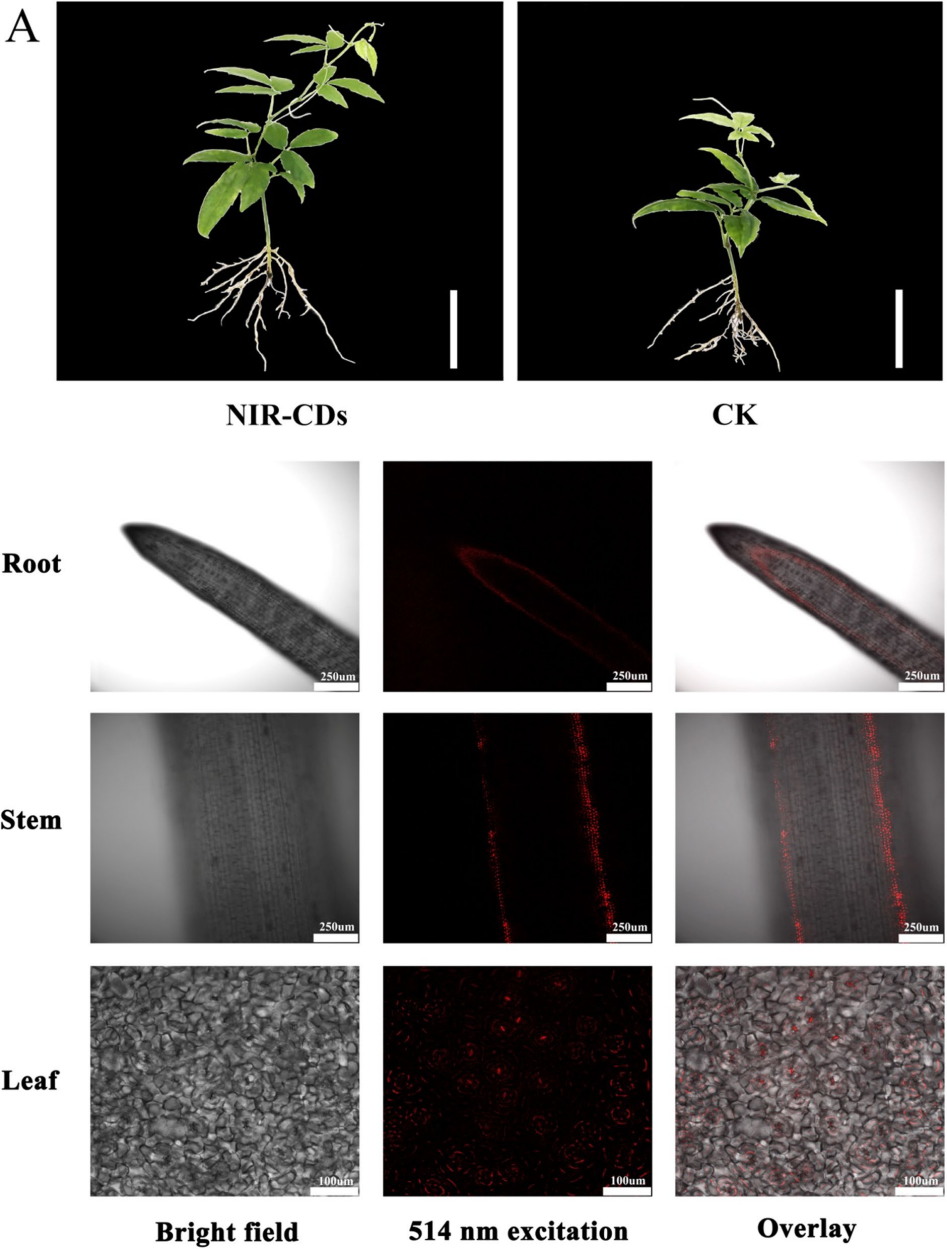

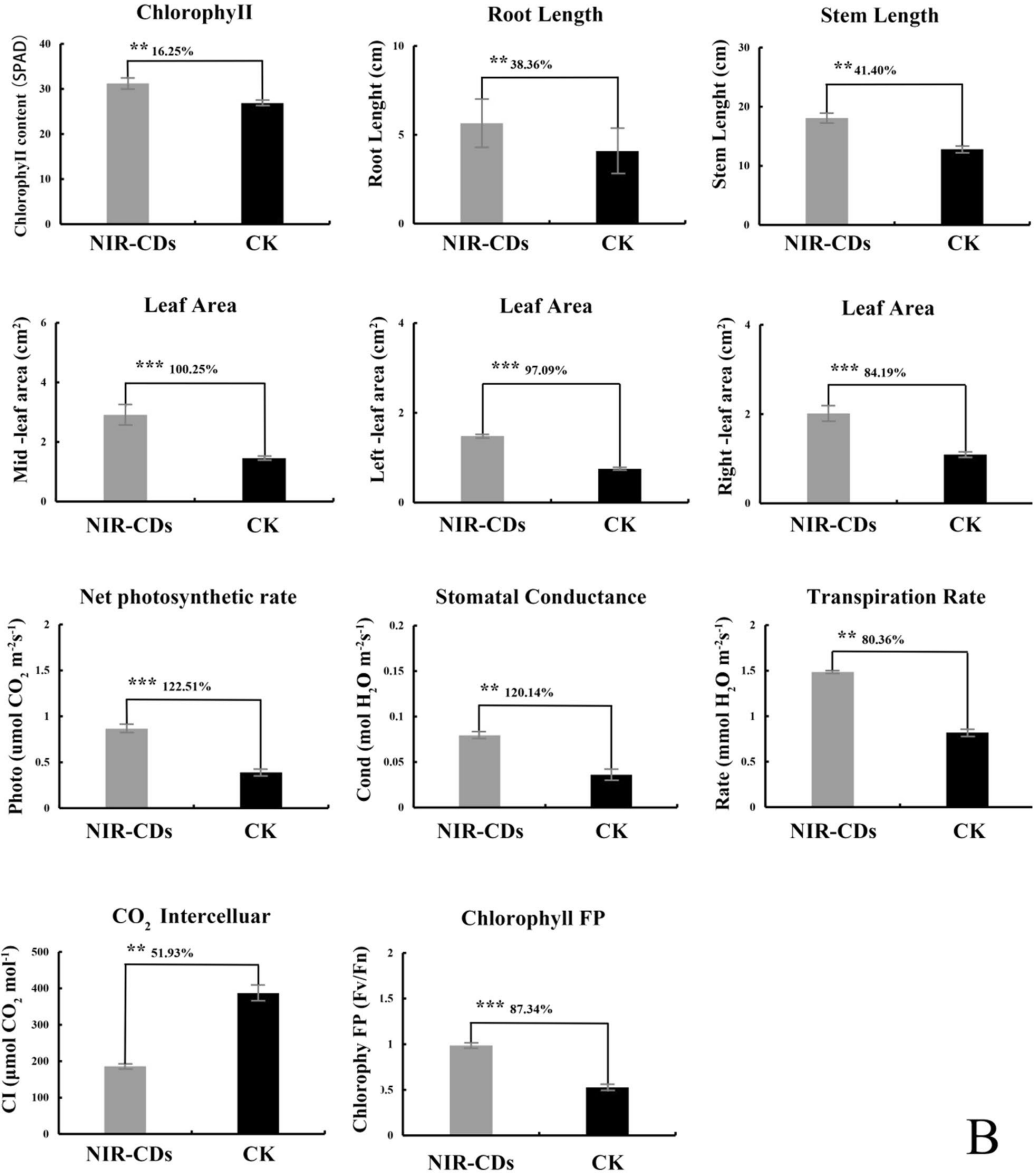
**A** The phenotypic and Laser scanning microscopy (LSM) images of root, stem, and leave cultured with NIR-CDs (0.05 mg/mL) (scale bar of phenotypic images: 10 cm; scale bar of laser scanning microscopy images: 250 μm). **B** Effect of NIR-CDs (0.05 mg/mL) on the growth potential indexes and photosynthetic parameters of *T. hemsleyanum* during 30 days. All the experiments were repeated three times at least. Marked with * (*P* < 0.05), ** (*P* < 0.01) and *** (*P* < 0.001) exhibit significant differences from control, respectively

Figure 3A is the phenotypic characteristic of *T. hemsleyanum* exposed to 0.05 mg/mL NIR-CDs on day 30. The treatment enables *T. hemsleyanum* to grow more exuberantly than the control group. As shown in Fig. 3B, compared to the control, root length, stem length, leaf area, net photosynthetic rate, stomatal conductance, transpiration rate, chlorophyll fluorescence parameter Fv/Fn and chlorophyll content were significantly increased by 38.36 ± 6.6%, 41.40 ± 1.9%, 93.84 ± 9.2%, 16.25 ± 1.2%,122.51 ± 10.6%, 120.14 ± 11.1%, 80.36 ± 7.1% and 87.34 ± 3.2%, respectively. Intercellular CO_2_ was decreased by 51.93 ± 3.8% than the control.

Confocal images were captured using Laser-scanning confocal fluorescence microscope after incubation with 0.05 mg/mL NIR-CDs. As shown in Fig. 3A, the red fluorescence signals from NIR-CDs observed under 514 nm excitation were widely distributed in root, stem, and leaf of *T. hemsleyanum*, which confirmed that NIR-CDs could penetrate cell wall into vascular bundle system, and were then transported to the aerial parts.

### Metabolome profiling and KEGG enrichment analysis of differential metabolites

**Fig. 4:**
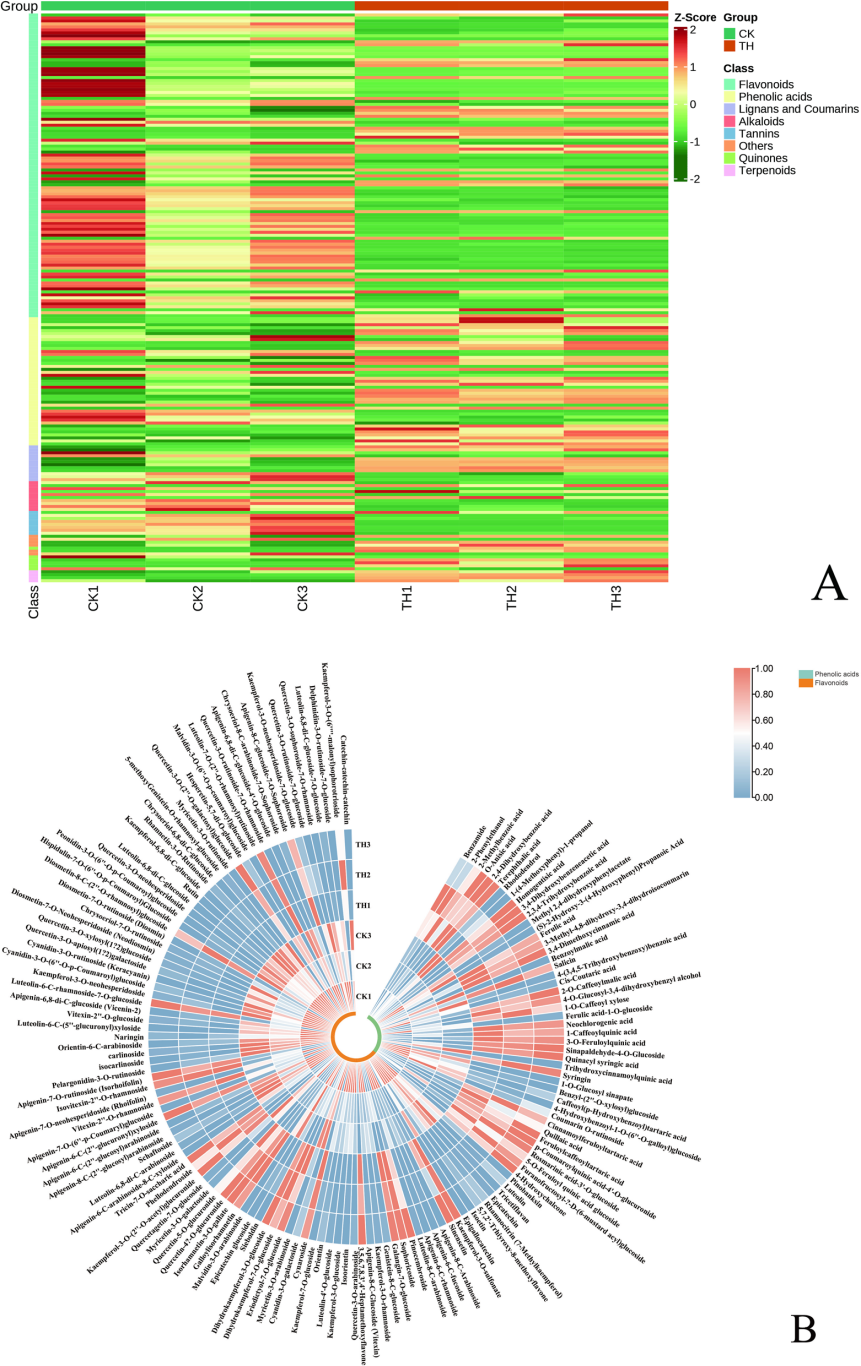
**A** The overall heatmap of 8 categories of secondary metabolites among CK and TH. **B** The heatmap of the fold changes of secondary metabolites among CK and TH of *T. hemsleyanum*. The color bar represents the normalized fold change values. Three categories of secondary metabolites were marked, including flavonoids (claybank), phenolic acids (nattier blue), and alkaloids/terpenoids (purple)

We profiled the widely-targeted LC–MS/MS based metabolome of the samples from the control (denoted as CK) and the NIR-CDs-treatment groups (denoted as TH), which represented the impact of the NIR-CDs on metabolite accumulation of *T. hemsleyanum*. Principal component analysis reflected the cluster grouping among the samples was favorable (Additional file 6: Fig. S6). We detected 493 secondary metabolites grouped into 8 classes (Additional file 11: Table S1), including 182 flavonoids, 167 phenolic acids, 20 lignans and coumarins, 64 alkaloids, 15 terpenoids, 26 tannins and others. The differentially accumulated metabolites (DAM) between the control and the experimental groups (CK_vs_TH) were screened using the variable importance in projection (VIP) ≥ 1 from the OPLS-DA model and fold change ≥ 1.5 (upregulated) or ≤ 0.667 (downregulated). A total of 191 DAM were identified in CK_vs_TH (Additional file 12: Table S2). These secondary metabolites can be mainly categorized into the classes of flavonoids and phenolic acids, including 106 flavonoids, 43 phenolic acids, 12 lignans and coumarins, 9 alkaloids, and others. Overall, flavonoids were more inclined to accumulate in CK than in TH. The content of phenolic acids, lignans and coumarins were significantly improved by CDs-treatment (Fig. 4A).

We focused on the two classes of secondary metabolites (flavonoids and phenolic acids) likely to be major contributors to biological activity (Fig. 4B). Flavonols and flavonoid carbonoside were identified with a series of glycoside derivatives of kaempferol, quercetin, and apigenin, which made up the majority of the DAMs detected for CK _vs_TH compared samples. Based on fold changes and VIP values, 77 out of 106 flavonoids were identified as down-accumulated significantly by CDs-treatment, the concentrations of Kaempferol-3-O-glucoside, Quercetin-3-O-neohesperidoside, Apigenin-6,8-di-C-glucoside-4'-O-glucoside, Quercetin-3-O-sophoroside-7-O-rhamnoside, Procyanidin C1, and Epigallocatechin were significantly greater in CK than in TH (Student’s t test, *P* < 0.05), and the concentrations of the remaining 29 flavonoids were significantly greater in TH than in CK. 43 out of 167 phenolic acids were identified as differentially accumulated by CDs-treatment, of these 13 phenolic acids were downregulated and 30 were upregulated in TH compared with CK, among which Quillaic acid, Furanofructosyl-α-D-(6-mustard acyl)glucoside, Feruloylcaffeoyltartaric acid were found only in TH, CDs-treatment also led to a tenfold enhancement in Syringin, Neochlorogenic acid, Homogentisic acid, and fivefold increase in 1-Caffeoylquinic acid and 2-Caffeoylquinic acid.

### Transcriptome analysis and DEGs identification

**Fig. 5:**
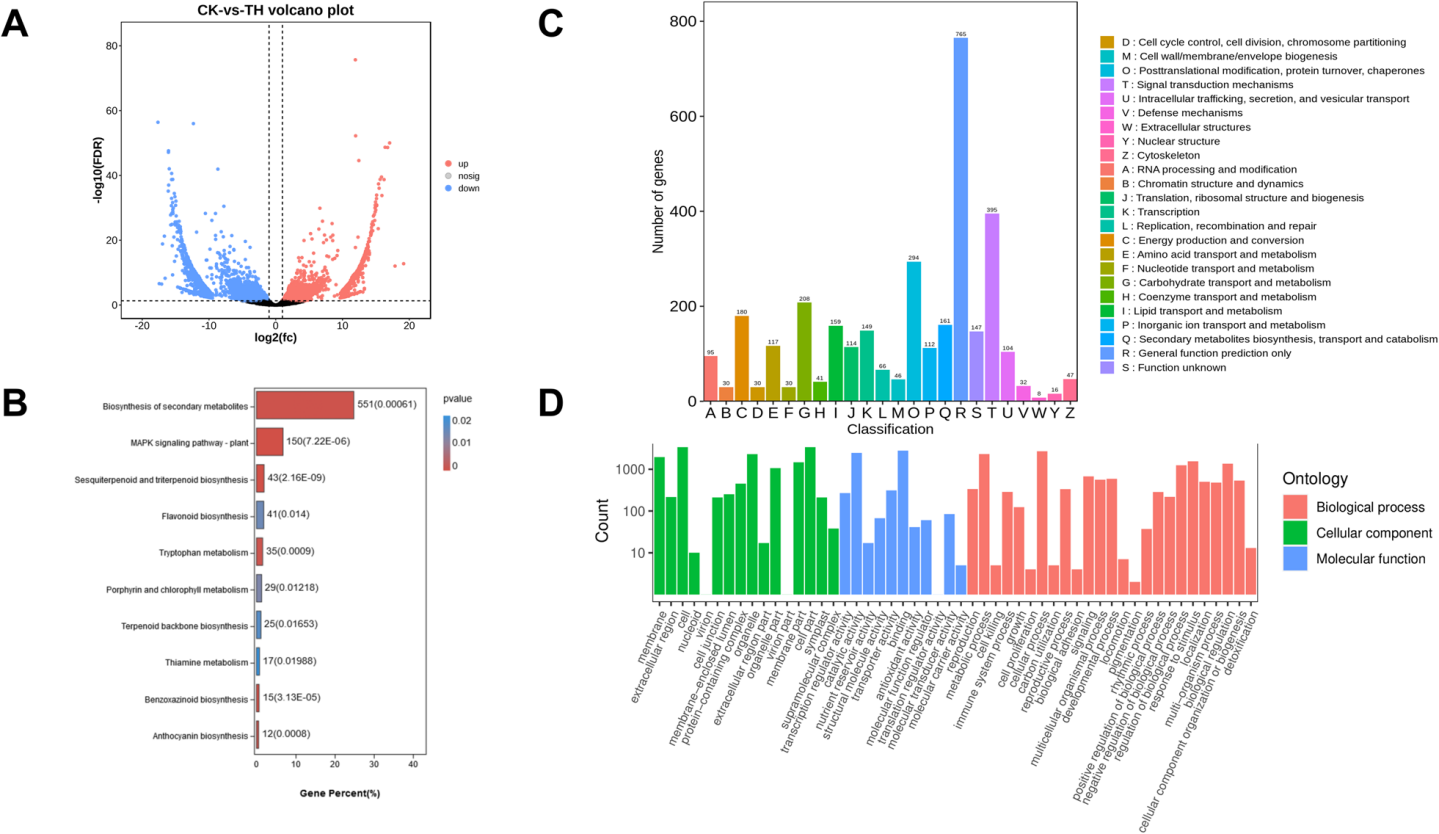
**A** Volcano plot of differential gene expression analysis. **B** KEGG pathway analysis for differential gene expression analysis. **C** KOG orthologous classification of differential gene. **D** GO level terms classification of differential genes

Differentially expressed genes (DEGs) in CK vs TH comparative group were identified by transcriptome analysis and genes identification. A total of 40.08–52.20 million clean reads were obtained, and the Q30 of the raw reads in all groups ranged from 91.08% to 92.21%. As shown in Fig. 5A, upregulated expressed genes (Log2FC ≥ 2) were marked as the red dots in volcano plot, downregulated expressed genes (Log2FC ≤ 0.5) and non-significantly differentially expressed genes (0.5 < Log2FC < 2) were marked as the blue dots and the black dots, respectively. The horizontal axis and the vertical axis indicate the fold change of differential expression and the significance level of the gene expression differences, respectively. According to the results of transcriptome analysis, 43,340 unigenes were identified. A total of 6874 DEGs, including 2962 upregulated genes and 3912 downregulated genes, were identified between CK vs TH (Additional file 13: Table S3).

The DEGs were subjected to KEGG, KOG, and GO functional pathway analyses. The top enriched KEGG terms contributed by these DEGs were ko01110 (biosynthesis of secondary metabolites), ko04016 (MAPK signaling pathway—plant), ko00941 (flavonoid biosynthesis), ko00860(porphyrin and chlorophyll metabolism), and ko00909 (sesquiterpenoid and triterpenoid biosynthesis) (Fig. 5B). The top enriched KOG terms included posttranslational modification, protein turnover, chaperones(O), Carbohydrate transport and metabolism(G), Energy production and conversion(C), and Secondary metabolites biosynthesis, transport and catabolism(Q) (Fig. 5C). DEGs annotated in GO functional pathway were divided into 52 functional groups, including 25 groups in biological process, 16 in cellular components, and 11 in molecular function. ‘Cell’, ‘Cell part’, and ‘organelle’ were the terms that dominated in the cellular component category. ‘catalytic activity’ and ‘binding’ predominated in the ‘molecular function’ category, ‘cellular process’, ‘metabolic process’, ‘biological regulation’, and ‘regulation of biological process’ were the most leading terms in the biological process category (Fig. 5D). The above analyses indicated that the identified DEGs were the most relevant to secondary metabolite metabolism and carbohydrate metabolism.

### Co-expression network analysis of DEGs

To identify the candidate genes regulating secondary metabolite metabolism and carbohydrate metabolism, an effective system biology method called weighted gene co-expression network analysis (WGCNA), was performed to find the modules of highly correlated genes and relate these modules to traits. Modules are clusters of genes with high correlation, and genes of a same module are co-expressed. A total of 8 gene modules were established on the clustering and signature analysis of the genes with similar expression patterns in DEGs. They were then used to correlate with the traits of flavonoid metabolites content (Additional file 7: Fig. S7), phenolic acids metabolites content (Additional file 8: Fig. S8), and photosynthetic efficiency (Additional file 9: Fig. S9), respectively. Soft threshold figures were showed as Additional file 10: Fig. S10. Among the 8 modules, the modules of “darkturquoise” and “green” were found to be associated with flavonoid, and phenolic acids accumulation. The “darkturquoise” gene module showed a strong correlation with the concentration of flavonoids, such as isorhamnetin-3-O-gallate (neohesperidin) (r = 1.0, *P* < 0.01), Quercetin-4′-O-glucuronide (r = 0.98, *P* < 0.01), Dihydrokaempferol-3-O-glucoside (r = 0.98, *P* < 0.01), Quercetin-3-O-(2''-O-galactosyl) glucoside (r = 0.98, *P* < 0.01), Dihydrokaempferol-3-O-glucoside (r = 0.98, *P* < 0.01), Luteolin-7-O-glucoside (Cynaroside) (r = 0.98, *P* < 0.01), Kaempferol-3-O-sulfonate (r = 0.92, *P* < 0.01), Apigenin-7-O-rutinoside (Isorhoifolin) (r = 0.97, *P* < 0.01), and so on. The “green” gene module also showed a high positive correlation with flavonoids, such as Kaempferol-3-O-glucoside (Astragalin) (r = 0.95, *P* < 0.01), Luteolin-8-C-glucoside (Orientin) (r = 0.98, *P* < 0.01), Apigenin-8-C-Arabinoside (r = 0.94, *P* < 0.05), and so on. The “darkturquoise” gene module showed a strong correlation with the concentration of phenolic acids, such as Syringin (r = 1.0, *P* < 0.01), 3,4-Dihydroxybenzeneacetic acid (r = 0.99, *P* < 0.01), Sinapaldehyde-4-O-Glucoside (r = 0.99, *P* < 0.01), 2-O-Caffeoylmalic acid (r = 0.93, *P* < 0.01), Neochlorogenic acid (r = 0.97, *P* < 0.01), Homogentisic acid (r = 0.94, *P* < 0.01), and so on.

The module–trait relationship analysis also identified module “darkturquoise” as most highly related to photosynthetic efficiency. Photosynthetic rate (Pn) (r = 0.97, *P* < 0.01), stomatal conductance (Cond) (r = 0.96, *P* < 0.01), transpiration rate (Tr) (r = 0.99, *P* < 0.01), chlorophyll fluorescence parameters (Fv/Fm) (r = 0.98,* P* < 0.01). Therefore, the “darkturquoise” and “green” modules were selected as the key gene modules for subsequent analysis.

### Key gene module expression and functional analysis

**Fig. 6:**
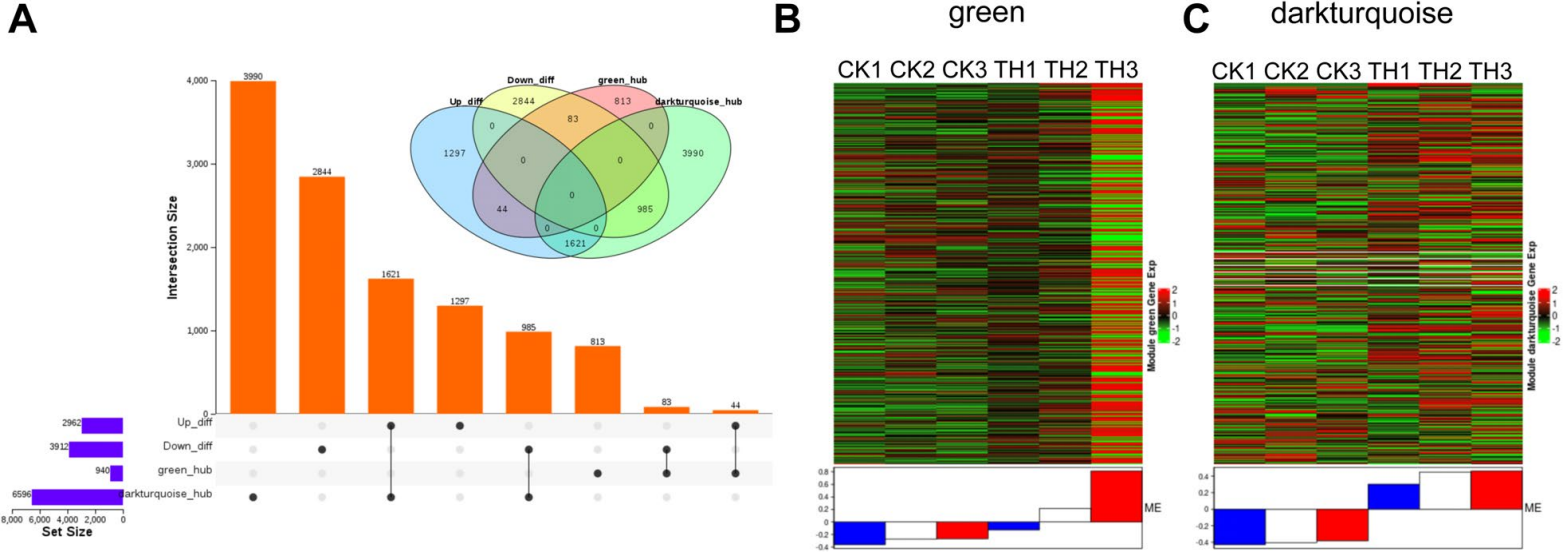
**A** Correlation Venn diagram of up-down regulated genes and phenotypic related gene module members. **B** Expression pattern of green module gene and column graphs of gene significance. **C** Expression pattern of darkturquoise module gene and column graphs of gene significance

Gene annotation and expression of all the gene members in the two key gene modules were performed. “darkturquoise” and “green”gene modules were composed of 6596 and 940 unigenes, respectively. When the differential genes of CK vs TH were separately intersected with the gene members of the two key gene modules, we found that the intersection was observed between the two key gene modules and the differentially expressed genes (Fig. 6A). Correspondence relation between gene modules and gene members was showed in Table S4. Downregulated expressed genes in “darkturquoise” and “green” gene modules were 985 and 83, while upregulated expressed genes in the two modules were 1621 and 44, respectively. Further expression analysis of gene members of the two key gene modules revealed distinct expression patterns in CK and TH (Fig. 6B and C).

**Fig. 7:**
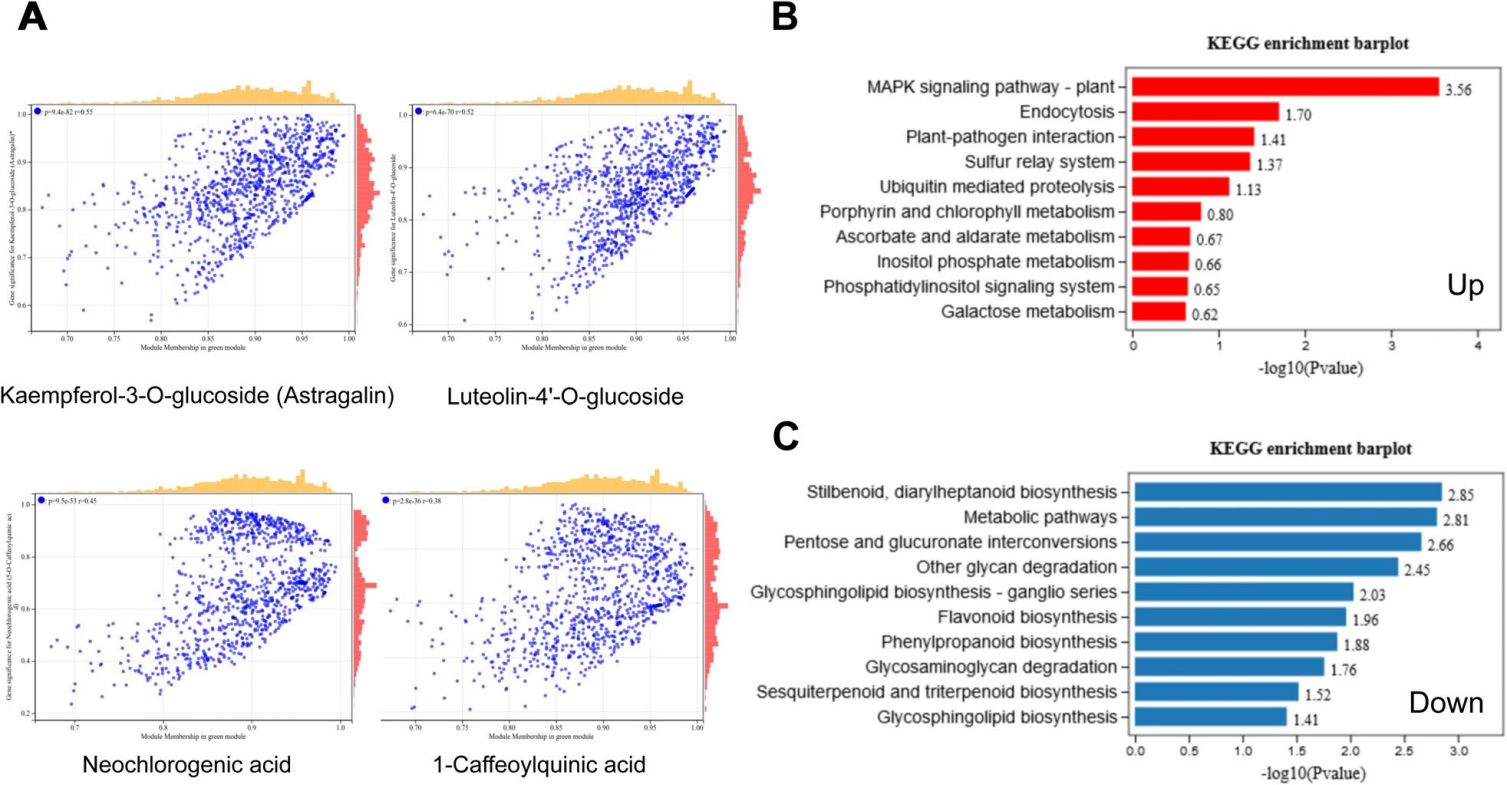
**A** Scatter plot of correlation between kaempferol-3-o-glucoside (astragalin), neogenetic acid (5-o-caffeoylquinic acid), 1-caffeoylquinic acid, luteolin-4'- O-glucoside and green module. **B** The top 10 enriched pathways of up-regulated genes in the green module by KEGG. **C** The top 10 enriched pathways of down-regulated genes in the green module by KEGG

**Fig. 8:**
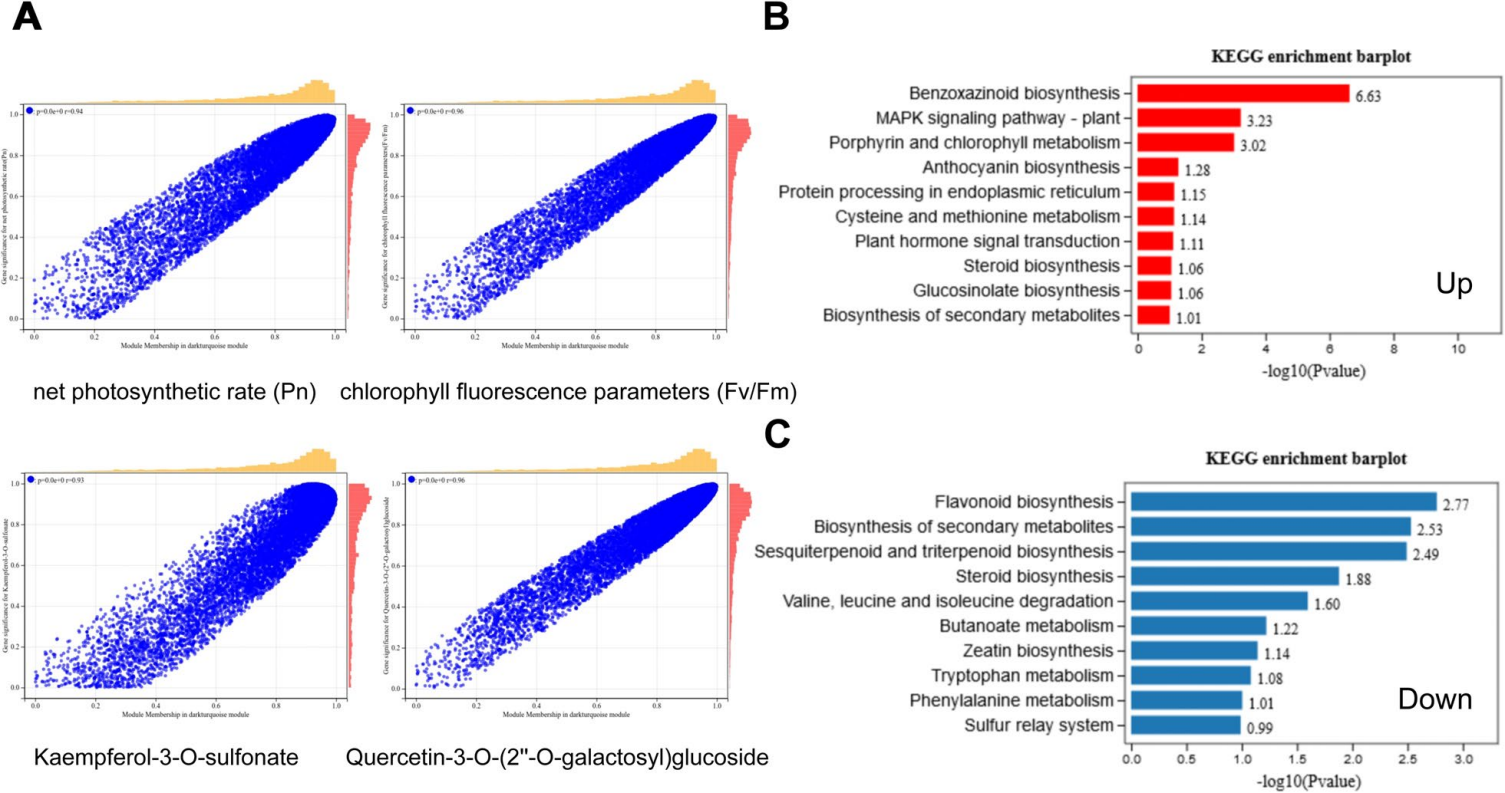
**A** Scatter plot of correlation between net photosynthetic rate (Pn), chlorophyll fluorescence parameters (Fv/Fm), kaempferol-3-O-sulfonate, quercetin-3-O-(2''-O-galactosyl) glucoside and darkturquoise module. **B** The top 10 enriched pathways of up-regulated genes in the darkturquoise module by KEGG. **C** The top 10 enriched pathways of down-regulated genes in the darkturquoise module by KEGG

The KEGG gene-set enrichment analysis of these DEGs in “green” and “darkturquoise” gene modules were severally shown as Figs. 7 and 8, respectively. For each screened gene module, the correlation scatter plot between some phenotypes and modules were drawn (Figs. 7A and 8A). The enriched KEGG terms of upregulated expressed genes in “green” gene module contained MAPK signaling pathway, porphyrin and chlorophyll metabolism, and galactose metabolism (Fig. 7B). The enriched KEGG terms of downregulated expressed genes in “green” gene module contained flavonoid biosynthesis, other glycan degradation, glycosaminoglycan degradation (Fig. 7C). The top enriched KEGG terms of upregulated expressed genes in “darkturquoise” gene module were benzoxazinoid biosynthesis, MAPK signaling pathway, porphyrin and chlorophyll metabolism, and anthocyanin biosynthesis (Fig. 8B). The top enriched KEGG terms of downregulated expressed genes in “darkturquoise” gene module were flavonoid biosynthesis, biosynthesis of secondary metabolites (Fig. 8C).

### Identification of hub genes within network modules

**Fig. 9:**
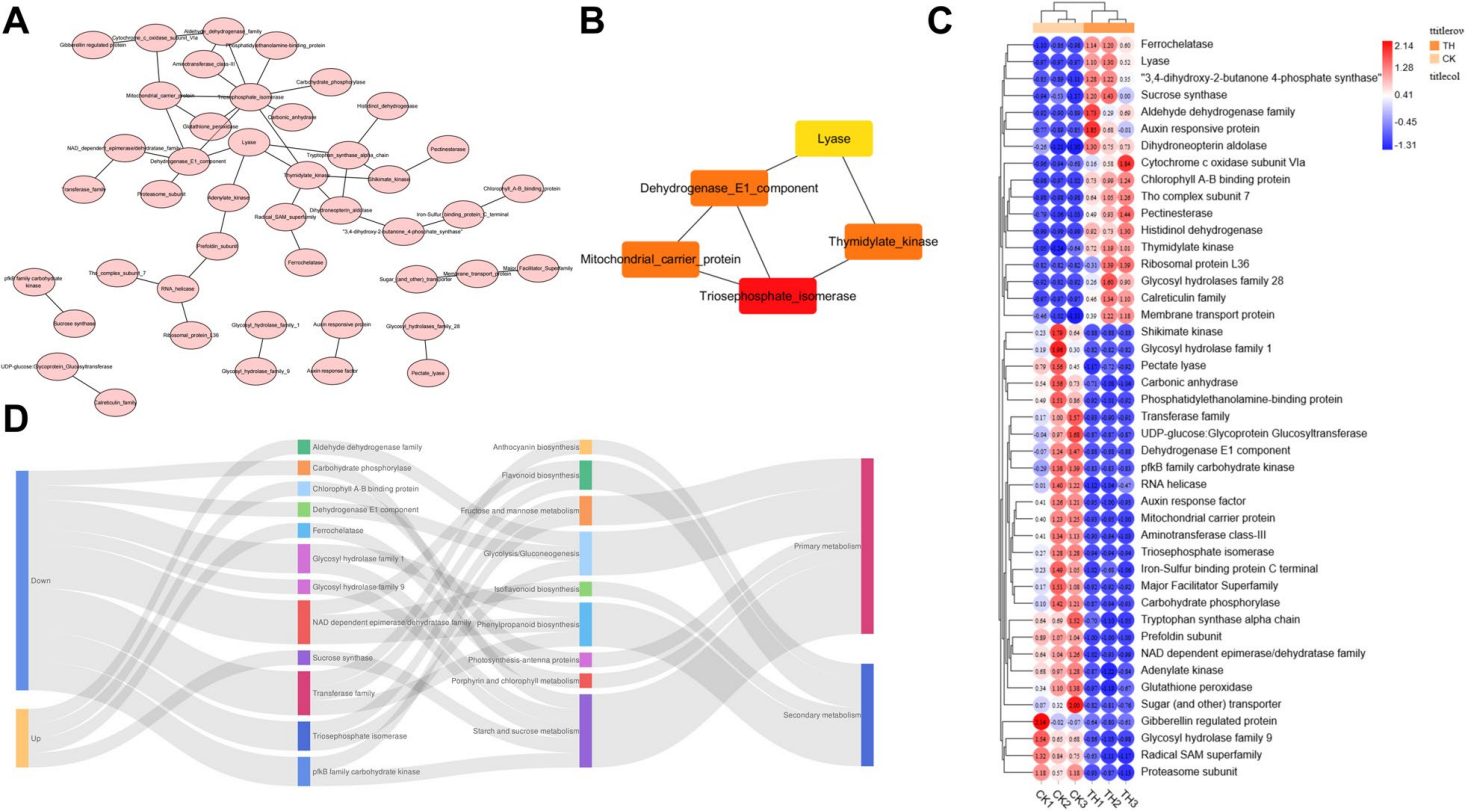
**A** Protein–protein interaction network. **B** The top 5 hub genes of screened the most important protein network nodes by MCC algorithm. **C** The expression pattern of node genes in PPI network. **D** The sanky plot of gene information in PPI network

Two modules, “darkturquoise” and “green”, were considered to take a decisive factor in the regulation of CDs on plant growth and secondary metabolites accumulation. Hub genes were defined as these genes which were highly associated with other genes in each module network, and played a central role within the network clusters. Hub genes within the two modules were discovered in Fig. 9. Protein–protein interaction (PPI) network, which took the gene members in the two key modules as its object, were obtained using string database, and a total of 44 nodes, 44 edges were identified (Fig. 9A). The MCC algorithm of CytoHubba was used to score and sort the hub nodes in the PPI network, and select the top 5 genes (triosephosphate isomerase, mitochondrial carrier protein, thymidylate kinase, dehydrogenase E1 component and lyase) as the hub genes of the PPI network (Fig. 9B). Gene expression of all PPI network gene members in the two key gene modules were performed and shown in Fig. 9C. Among them, 17 up-regulated genes and 27 down-regulated genes were identified. These interactions among key genes, pathways, metabolic type and gene regulation are shown in Fig. 9D. As for secondary metabolism, transferase family genes in anthocyanin, phenylpropanoid, and flavonoid biosynthesis pathways tended to be down-regulated after CDs-treatment, and the expression of NAD dependent epimerase/dehydratase family genes in phenylpropanoid, flavonoid, and isoflavonoid biosynthesis pathways were restrained as well. As for primary metabolism, some gene families of carbohydrate metabolism, such as pfkB family carbohydrate kinase, glycosyl hydrolase family, triosephosphate isomerase, and carbohydrate phosphorylase, tended to be down-regulated after CDs-treatment. However, some gene families involved in carbohydrate synthesis, such as sucrose synthase and aldehyde dehydrogenase families, were up-regulated. Ferrochelatase and chlorophyll A–B binding protein, which were considered to be vital components of photosynthesis and chloroplast synthesis, were both up-regulated in response to CDs-treatment.

### Validation of hub genes expression by qRT-PCR

**Fig. 10:**
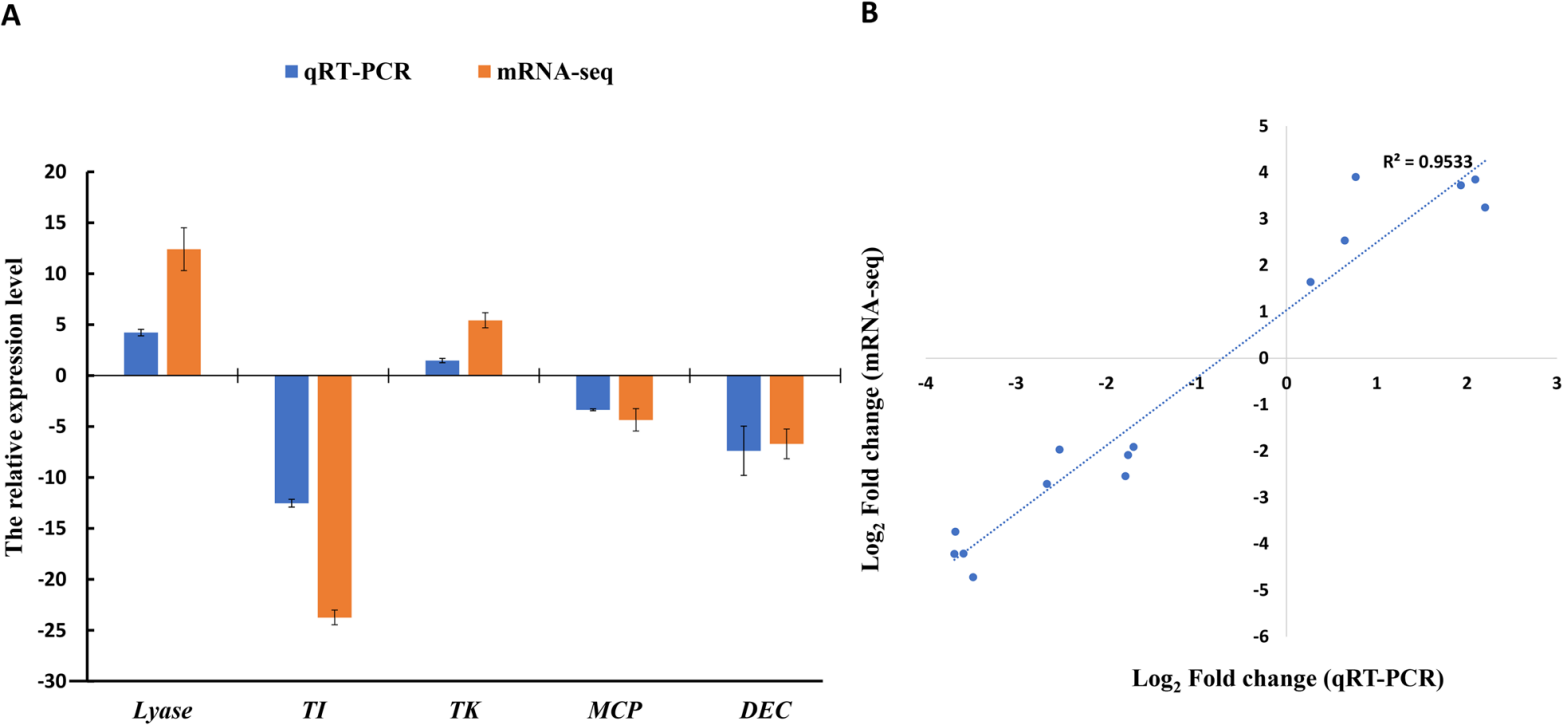
**A** Verification of RNA-Seq sequencing data by the qRT-PCR assay. Lyase, Triosephosphate isomerase (TI), Thymidylate kinase (TK), Mitochondrial carrier protein (MCP), Dehydrogenase E1 component (DEC). **B** Pearson correlation coefficients of the log2 of gene expression ratios obtained from RNA-seq data and qRT-PCR. All qPCR data were collected from three biological replicates and three technical replicates for each sample

We selected the top 5 hub genes (triosephosphate isomerase, mitochondrial carrier protein, thymidylate kinase, dehydrogenase E1 component and lyase) of the PPI network to conduct a validation experiment by qRT-PCR analysis. The expression patterns of RNA-Seq of all the 5 hub genes were highly consistent with the qRT-PCR analyse, and the correlation coefficient (R2) was 95.33% (Fig. 10). Both qRT-PCR and RNA-seq results showed that three hub genes (triosephosphate isomerase, mitochondrial carrier protein, and dehydrogenase E1 component) showed lower level of expression after CDs-treatment. However, other two genes, thymidylate kinase and lyase had much higher expression rate in the CDs-treatment group.

## Discussion

As an emerging research hotspot in nanotechnology, carbon dots (CDs) have attracted much attention. Due to the fluorescence property of CDs, it is available to trace the uptake and accumulation of CDs in plants. When the mung bean was treated with nitrogen-doped CDs by solid-state method, the blue fluorescence was observed to migrate from the beans to the root ends by ultraviolet light during the sprouting process [40]. When *T. hemsleyanum* was treated with red CDs, the fluorescence signal of CDs was mainly detected in the vascular system of the root, stem, and leaf by confocal laser scanning microscope in our study, which confirmed that CDs could easily cross the biological barrier and be widely distributed in the plants. A number of studies have demonstrated that CDs can promote the growth of various plants by enhancing the light absorption [11], stimulating biosynthase activity [41], converting ultraviolet light into blue and red light [17, 18], and we firstly demonstrated that potential molecular mechanisms behind the growth-stimulating effect might be related to up-regulated expression of the primary metabolism related genes, among which *PsbP* and *PsiK* genes were the hub genes [23].

Plants synthesize a wide variety of secondary metabolites that are derived from central or primary metabolism. Flavonoids, alkaloids, terpenoids and phenolic acids are the common and important kinds of secondary metabolites, and they are closely related to multiple bioactive functions of medicinal plant. It is generally assumed that the activity level of primary metabolism would have a major positive impact on the growth of plant and the level of secondary metabolism would be stimulated under environmental stress [42]. Hence, it is of great significance to investigate the potential impact of CDs on the metabolic transition between primary and secondary metabolism.

*T.hemsleyanum* is a herbal plant distributed in tropical to subtropical regions, mainly in mainland China. Flavonoids and phenolic acids were the main active secondary metabolic ingredients of *T. hemsleyanum* [43]. Total favonoids from *T. hemsleyanumon* were reported to exert anti-infammatory effects on Con A-induced hepatitis in mice [44]. *T. hemsleyanum* flavonoids could inhibit the migration and promote the apoptosis of A549 cells both in vitro and in vivo [45]. Phenolics in *T. hemsleyanum*, including 1-Caffeoylquinic acid, 2-Caffeoylquinic acid, 5-p-coumaroylquinic acid, isoorientin etc., were related to the antioxidant and antiproliferative activities of *T. hemsleyanum* [46]. A total of 13 bioactive compounds, including kaempferol, caffeic acid, apigenin, quercetin etc., were considered to be related to alleviating lung infection in *Pseudomonas aeruginosa*-induced mice [47]. Apigenin and luteolin glycosides of *T. hemsleyanum* were reported to induce apoptosis in HepG2 cells as well as inhibit the tumor growth in H22 tumor-bearing mice [48].

In this study, CDs-treatment had the opposite effect on the content of phenolic acids and flavonoids. The contents of flavonoids decreased and phenolic acids substance increased. The ingredients associated with anti-inflammatory and antioxidant activities of *T. hemsleyanum*, such as syringin, neochlorogenic acid, homogentisic acid, 1-Caffeoylquinic acid and 2-Caffeoylquinic acid, were generally improved in response to CDs-treatment. But the ingredients associated with anti-tumor activity, such as glycoside derivatives of kaempferol, quercetin, and apigenin, were more likely to decrease in general terms. Thereafter, we carried out an integrated transcriptome and metabolome research on the regulatory networks of primary and secondary metabolites biosynthesis, including starch and sucrose metabolism, photosynthesis, flavonoids biosynthesis etc. of *T. hemsleyanum* under the treatment of NIR-CDs. The pattern of association between differentially expressed genes (DEGs) and metabolite components were explored by WGCNA, then 44 candidate hub genes highly related to quality ingredients accumulation and growth were identified.

Maintenance of deoxyribonucleotide levels plays a major role in ensuring genome stability of DNA replication and biosynthesis. The increase in the deoxyribonucleotide pools would lead to functional changes in DNA Pol III leading to more elongation and less proof-reading activity, thus resulting in the activation of translesion synthesis. Thymidylate kinase is an important enzyme in DNA synthesis and catalyses the conversion of dTMP to dTDP. In the present study, the activity of thymidylate kinase was significantly stimulated by NIR-CDs-treatment, serving as one of the top 5 hub genes. It suggested that thymidylate kinase gene was highly associated with other genes in DNA synthesis and cell division gene regulatory network, and played a central role in promoting primary metabolism of *T. hemsleyanum*. Thymidylate kinase has also been studied in the photosynthetic nitrogen-fixing cyanobacterium Nostoc. Recombinant Nostoc strains overexpressing AnTMK exhibited higher growth rate measured in terms of chlorophyll *a* content under normal growth conditions, which indicated that the TMK is likely to have a significant role in photosynthetic organisms [49].

Mitochondrial carrier family (MCF) proteins of plant are indispensable for transporting TCA-cycle intermediates across the inner mitochondrial membrane [50]. The present study also suggested MCF served as a protagonist in the regulation of mitochondrial metabolism in response to CDs-treatment. Chlorophyll and chlorophyll A–B binding protein are crucial for the assembly of a functional photosystem II, which is responsible for light absorption, excitation energy transfer, and charge separation. Ferrochelatase in plants possesses a conserved transmembrane chlorophyll A–B binding domain that is somewhat analogous to the first and the third helix of light-harvesting complexes, including a chlorophyll-binding motif [51]. In the present study, CDs-treatment significantly stimulated photosynthesis of *T. hemsleyanum* by simultaneously upregulating the expression of chlorophyll A–B binding protein and ferrochelatase.

Glycosyl hydrolase family genes are involved in many aspects of plant physiological processes, in particular response to environmental stresses through the regulation of phytohormones and defensive components. Four genes of the glycoside hydrolase family 1 in the model legume plant *Medicago truncatula*, were dramatically activated by NaCl, PEG, IAA, ABA, SA and GA3 treatments [52]. Both triosephosphate isomerase and pfkB family carbohydrate kinase are key enzymes in glucolysis [53]. Sucrose synthase plays an important role in tricarboxylic acid cycle and polysaccharides synthesis. In the present study, CDs-treatment significantly affected glucose metabolism by simultaneously inhibiting triosephosphate isomerase and pfkB family carbohydrate kinase, while activating sucrose synthase, which suggested that CDs could switch the glycometabolism flow from the glucolysis pathway to the pyruvate carboxylation, and hence the generation of polysaccharide would be improved.

Transferases are a superfamily of abundant enzymes that play vital roles in plant growth and development and flavonoid metabolism. For example, UDP-glycosyltransferases were essential for flavonoid biosynthesis in *Glycyrrhiza uralensis* [54]*.* A glutathione *S*-transferase gene was demonstrated to be a candidate gene for regulating anthocyanin accumulation and pigmentation in cotton tissues [55]. Transferases are likely to be responsive to exogenous chemical ingredients or biotic and abiotic stresses, and thus the accumulation of plant-derived flavonoids was affected. The glutathione transferases had members that are selectively induced by chemical stress treatments, which were confirmed to play vital roles in redox homeostasis and maintaining the flavonoid pool [56].

## Conclusion

In summary, UV-absorptive and water-soluble NIR-CDs were harvested via a facile microwave-assisted carbonization method. The NIR-CDs were successfully adopted for enhancing photosynthesis and growth of *T. hemsleyanum*, and the influences on the accumulation of bioactive ingredients was illustrated as well. A total of 191 differential secondary metabolites and 6874 differentially expressed genes were identified. The phenolic acids were generally improved, but the flavonoids were more likely to decrease in general terms. The pivotal differentially expressed genes were involved in multiple metabolic processes related to biosynthesis of secondary metabolites, flavonoid biosynthesis, porphyrin and chlorophyll metabolism, etc. Subsequently, DEGs were divided into 9 modules by WGCNA. Two modules positively correlated with the flavonoids content, phenolic acids content and photosynthetic efficiency were identified, in which 44 hub genes and the top 5 genes of the PPI network were identified. This research provided key regulatory genes and differentially accumulating quality ingredients under CDs-treatment, which could provide a theoretical basis for expanding the applications of nanomaterial in industrial crop agriculture.

## Materials and methods

### Materials and apparatus

Reduced glutathione (GSH), Na_2_HPO_4_, KH_2_PO_4_ and KCl were purchased from Aladdin Chemistry Co., Ltd (Shanghai, China). Formamide was obtained from Sinopharm Chemical Reagent Co., Ltd. (Shanghai, China). All of the chemicals were used as received without further purification. Aqueous solutions were prepared using deionized (DI) water.

A microwave oven (Galanz, P70F20CL) was adopted for the fabrication of NIR-CDs. The sizes and morphologies of the obtained NIR-CDs were characterized by high resolution transmission electron microscopy (HR-TEM, Tecnai F20) using an acceleration voltage of 200 kV. Fourier transform infrared (FT-IR) spectrum was carried out on a Nicolet 6700 FT-IR spectrometer through the KBr pellet technology. Raman spectrum was recorded on a Renishaw inVia Raman spectrophotometer via a 532 nm laser as the excitation resource. The crystal phase of NIR-CDs was measured on a Bruker D8 Discover X-ray diffractometer (XRD) with 2θ range from 10° to 50° at a scanning rate of 4°/min, with Cu Ka irradiation (k = 1.5406 Å). Fluorescence spectra were measured on a Perkin Elmer spectrophotometer (LS-55). UV–vis absorption spectra were recorded on an Agilent Cary 300 spectrophotometer. Fluorescence delay curve was monitored by Fluorolog 3–11 (HORIBA Jobin Yvon). Absolute fluorescence quantum yield (QY) was directly measured by a Fluoromax-4 measurement system (HORIBA, JobinYvon. Inc).

### Synthesis of NIR-CDs

NIR-CDs were synthesized in accordance with our previously-reported one-pot microwave-assisted carbonization manner [23, 57]. Briefly, reduced GSH (2.0 g) was dissolved in 20 mL formamide under a 10 min treatment of ultrasonic. Thereafter, the mixture was transferred into a domestic microwave oven for further carbonization reaction (700 W, 3 min). Then, the beaker was cooled down to room temperature naturally, and a dark green mixture was obtained. The mixture was centrifuged at 10,000 rpm for 5 min to remove large-sized nanoparticles, and purified through dialysis (cut-off molecular weight, 3500) for 5 days. Then, the NIR-CDs solution was concentrated and dried using a rotary evaporation. Finally, the dark green NIR-CDs powder ca. 350 mg was harvested.

### Cytotoxicity testing of the NIR-CDs

A routine MTT assays against 16-HBE and MCF-7 cells were performed to assess the cytotoxicity of the NIR-CDs. In brief, the selected cells were cultured in a 96-well plate (1 × 10^5^ cells per mL) and adhered overnight. After a incubation of 24 h at 37 °C, the cells were then treated with Dulbecco's Modified Eagle's Medium (DMEM) containing various concentrations of NIR-CDs (0–200 μg/mL) for another 24 h cultivation. Then, the culture medium was removed and 10 μL MTT (5.0 mg/mL in PBS) was added into each well for additional 4 h incubation. Subsequently, 100 μL of DMSO was added to dissolve MTT. Finally, the optical absorption intensity of each sample was recorded by a microplate reader.

### Plant cultivation and NIR-CDs treatment

Healthy cutting seedlings of *T. hemsleyanum* were cultured in a greenhouse under a 16/8 h photoperiod (day/night), 23 ± 2 °C temperature, and 50% relative humidity. Then, seedlings showing consistent growth state were selected and uniformly divided into two groups, CK_and_TH. NIR-CDs solutions at 0.05 mg/mL were sprayed on seedlings with a dosage of 10 mL/pot every day (TH), and the same volume of ultrapure water was sprayed as the control (CK). Phenotypic changes were observed and recorded every 15 days, respectively. The photosynthetic parameters were determined according to our previous report [58]. Each treatment was repeated three times with ten plants. The young leaves were collected on 30 nd day, frozen in liquid nitrogen immediately, and stored at −80℃ for RNA-seq and metabolomics analysis. Three biological replicates were performed, and each biological replicate consisted of a pool of samples from 10 seedlings.

### RNA-seq data analysis

RNA extraction, quantification and transcriptome sequencing were performed according to previous studies [59, 60]. Sequencing libraries were generated using NEBNext® UltraTM RNA Library Prep Kit for Illumina® (NEB, USA). The clustering of the index-coded samples was performed on a cBot Cluster Generation System using TruSeq PE Cluster Kit v3-cBot-HS (Illumia). The library preparations were sequenced on an Illumina Hiseq platform and 125 bp/150 bp paired-end reads were generated. De novo Transcriptome was assembled using Trinity software. The assembled transcript sequence and KEGG, NR, Swiss-Prot, GO, KOG, Trembl databases were compared using DIAMOND BLASTX software [61]. After predicting the amino acid sequence of the transcript, HMMER software was used to compare with Pfam database to obtain the annotation information. The gene expression quantity calculations were performed by bowtie2 [62]. Fragments per kilobase of transcript per million mapped reads (FPKM) of each gene based on the gene length were used to calculate the gene expression level.

The differential expression among the groups was compared using edgeR. The corrected P value and |log2foldchange| are used to evaluate significant difference expression (log2|FoldChange|> 2 and P value < 0.05 were adopted as the screening threshold). The enrichment analysis is performed based on the hypergeometric test. Kyoto Encyclopedia of Genes and Genomes (KEGG) were adopted as pathway enrichment. At least three genes were enriched in pathway, and a p value less than 0.05 was considered to be significantly enriched.

### Extraction, qualitative and quantitative analysis of metabolites

Dissolve 100 mg of freeze-dried sample powder with 1.2 mL 70% methanol solution, the mixture was extracted overnight at 4 ℃ and then analyzed using an UPLC-ESI–MS/MS system (UPLC, SHIMADZU Nexera X2, https://www.shimadzu.com.cn/; MS, Applied Biosystems 4500 Q TRAP, https://www.thermofisher.cn/cn/zh/home/brands/applied-biosystems.html), which was equipped with Agilent SB-C18 UPLC column (1.8 µm, 2.1 mm*100 mm). The mobile solvent system was composed of solvent A, water (0.1% formic acid), and solvent B, acetonitrile (0.1% formic acid). The gradient program was 95:5 V/V at 0.0 min, a linear gradient to 5% A, 95% B was programmed within 9 min, and then 5: 95 V/V was kept for 1 min. Flow rate and injection volume was 0.35 mL/min and 4 µL, respectively.

Linear ion trap (LIT) and triple quadrupole (QQQ) scans were acquired on an AB4500 Q TRAP UPLC/MS/MS System, equipped with an ESI Turbo Ion-Spray interface, and controlled by Analyst 1.6.3 software (AB Sciex). The ESI source operation parameters were as follows: ion source, turbo spray; ion spray voltage (IS) 5500 V (positive ion mode)/-4500 V (negative ion mode); source temperature 550 °C; ion source gas I (GSI), gas II(GSII) and curtain gas (CUR) were fixed at 50, 60, and 25.0 psi, respectively. Instrument tuning and mass calibration were performed with 10 and 100 μmol/L polypropylene glycol solutions in QQQ and LIT modes, respectively.

Metabolites were identified according to the m/z values, retention time, and fragmentation patterns of the standards in a database of MetWare Biotechnology Co., Ltd. (Wuhan, China). The filtering conditions for differentially accumulated metabolites were determined by VIP ≥ 1 and Log2FC (fold change) ≥ 1. VIP values were extracted from OPLS-DA result.

### Weighted gene co-expression network analysis (WGCNA)

Weighted gene co-expression network analysis (WGCNA) is a systems biology method used to describe the gene association patterns between different samples. It can be used to identify highly synergistic gene sets, and identify functional gene sets that affect or participate in phenotypes according to the interconnection of gene sets and the association between gene sets and phenotypes [63]. R software WGCNA was used to construct an undirected co-correlation network, defining a soft threshold β = 1 for the screening condition. The minimum number of module genes was 30 and the module merging threshold was 0.25, the cluster tree was constructed according to the correlation of gene expression and divided into modules. In order to identify gene modules and gene module members related to flavonoids and phenolic acid metabolism, the contents of net photosynthetic rate (Pn), stomatal conductance (Cond), transpiration rate (TR), intercellular carbon dioxide concentration (CI), chlorophyll fluorescence parameters (Fv/FM), the trait matrix of related phenotypes were used to calculate the correlation between gene module and phenotype. The expression patterns of hub module gene members were quantitatively described by intramodular gene expression heatmap with gene significance analysis. The phenotype and gene significance of the screened gene modules were analyzed, and the Venn plot was used to describe the up-down relationship of differential genes and the correlation between phenotypic related module gene members, so as to determine the signal pathways and metabolic pathways that are inhibited or stimulated in KEGG enrichment analysis and identification of gene modules.

### Identification of hub genes associated with secondary metabolite synthesis

Using the functionally annotated differential transcript data, the screened modules related to phenotype are combined to generate node information for constructing protein–protein network. Then, the near source species of *T. hemsleyanum* was selected for protein–protein interaction (PPI) analysis through STRING database (https://www.string-db.org/). Use Cytoscape software to draw the PPI network, then use the MCC algorithm of CytoHubba plug-in to score and sort the key nodes in the PPI network, and select the top 5 genes as the hub genes of the PPI network. Then, through the cluster analysis of the expression pattern of node genes in PPI network, the expression heatmap of these network member genes was drawn. Finally, the gene information in this network was described by sanky plot.

### Validation of the DEGs data using qRT-PCR

Expression levels of selected hub genes were analyzed by Quantitative real-time PCR (qRT-PCR) using CFX96 real-time PCR system (BIO-RAD, USA). Total RNA of *T. hemsleyanum* was isolated using Plant Total RNA Isolation Kit (Sangon, Shanghai, China). cDNA synthesis was performed with TransScript® Reverse Transcriptase (TransGen, Beijing, China). Each PCR reaction system contained 10 μl of 2 × Power SYBR green PCR master mix (Applied Biosystems, Forster City, CA, USA), 2 µl of template cDNA, 2 μl of forward and reverse primers (50 pmol) in a final volume of 20 µl. The PCR reactions were conducted by incubation at 95 °C for 3 min followed by 40 cycles of 95 °C for 15 s and 60 °C for 40 s. The actin gene was served as the internal reference. Transcripts were calculated by the 2^−ΔΔCt^ method. Real-time primers were presented in Table 1. Each gene was tested in triplicate with three biological replicates. The data represents the mean ± SD of three independent experiments.

**Table 1:** List of primer sequences of top hub genes used for qRT-PCR analysis

Gene annotation	Gene ID	Primer F (5’-3’)	Primer R (5’-3’)	Tm	Length(bp)
Lyase	Cluster-9600.25525	AGAGGTTCCACAGTTCAG	CAAGCACCTTAGCAATCTC	60	182
Mitochondrial carrier protein	Cluster-9600.54068	GTGGTCTTCCTCTTCGTAT	TGGTAGGAGCAGGAGTAA	60	123
Thymidylate kinase	Cluster-9600.8465	ACCGTTACTCCTATTCTGG	TCTCTGGTGGTATGTCAAG	60	126
Dehydrogenase E1 component	Cluster-9600.77806	GCGAGAACAATCACTATGG	AGCATCCATACCATCTACC	60	113
Triosephosphate isomerase	Cluster-9600.42222	TTAGCCTATGAGCCTGTG	ACTAACGGAACCTCCATAC	60	156

## Supplementary Information

**Additional file 1: Fig. S1**. XRD pattern (a) and Raman spectrum (b) of the obtained NIR-CDs.

**Additional file 2: Fig. S2**. High resolution XPS spectrum of C 1 s (a), N 1 s (b), O 1 s (c) and S 2p (d), respectively.

**Additional file 3: Fig. S3**. Zeta potential measurement of the NIR-CDs.

**Additional file 4: Fig. S4**. Fluorescence delay curve of the NIR-CDs solution (20 µg/mL, λ_ex_: 450 nm, λ_em_: 670 nm).

**Additional file 5: Fig. S5**. Cytotoxicity assessment of the NIR-CDs via the standard MTT assay toward 16HBE and MCF-7 cells.

**Additional file 6: Fig. S6.** Clustering relationship between gene modules.

**Additional file 7: Fig. S7.** Correlation heatmap between flavonoids content and module.

**Additional file 8: Fig. S8.** Correlation heatmap between phenolic acids content and module.

**Additional file 9: Fig. S9.** Correlation heatmap between photosynthetic efficiency and module.

**Additional file 10: Fig. S10.** Soft threshold figures for the weighted gene co-regulatory network analysis.

**Additional file 11: Table S1.** All secondary metabolites identified in *T. hemsleyanum*.

**Additional file 12: Table S2.** All differentially accumulated metabolites identified between CK vs TH.

**Additional file 13: Table S3.** All differentially expressed genes identified between CK vs TH.

**Additional file 14: Table S4.** Correspondence relation table between gene modules and gene members.

## Data Availability

Complementary data of NIR-CDs are supplied as Additional files 1, 2, 3, 4, 5: Figs. S1–S5. The metabolome and transcriptome original datasets generated in the current study are available in Additional files 11, 12, 13: Table S1–S4. Co-express network construction information generated in the current study are available in supplementary files Additional files 6, 7, 8, 9, 10: Figs. S6–S10.
