# Supplementary figures and images for: Multi-omics analyses revealed key factors involved in fluorescent carbon-dots-regulated secondary metabolism in Tetrastigma hemsleyanum
Source: J Nanobiotechnology. 2022 Feb 2;20:63. doi: 10.1186/s12951-022-01271-6 (PMC8812181; doi:10.1186/s12951-022-01271-6)

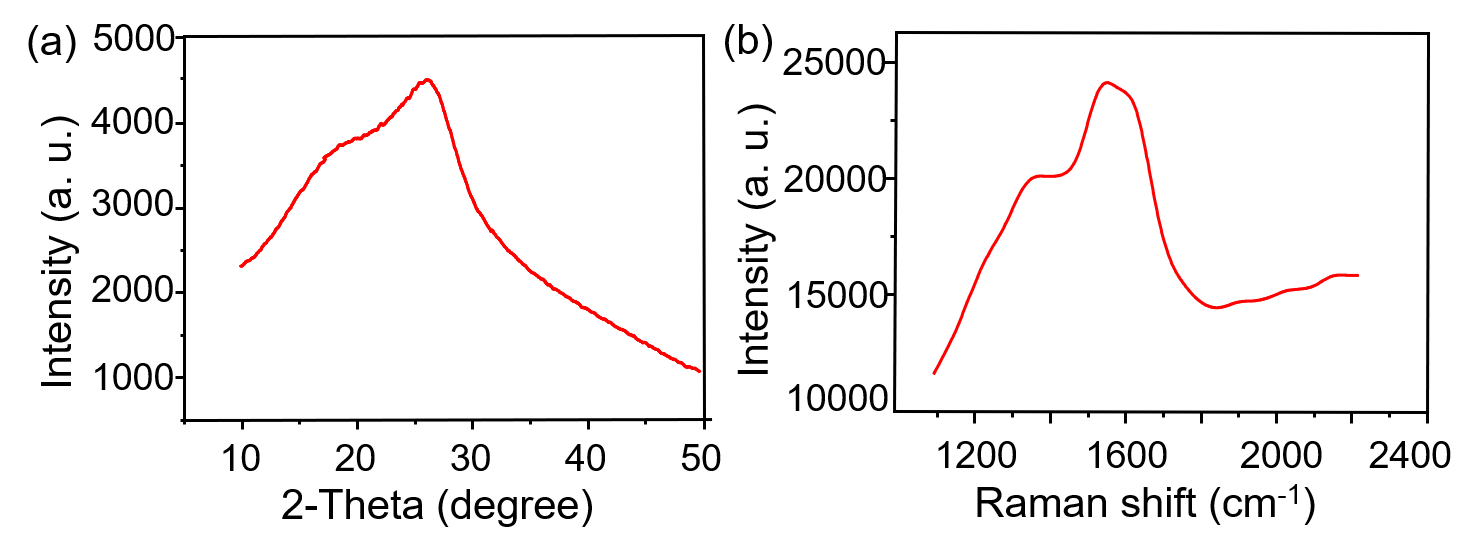

Supplement: Supplementary file 1 — Additional file 1: Fig. S1. XRD pattern (a) and Raman spectrum (b) of the obtained NIR-CDs. [file 12951_2022_1271_MOESM1_ESM.png]

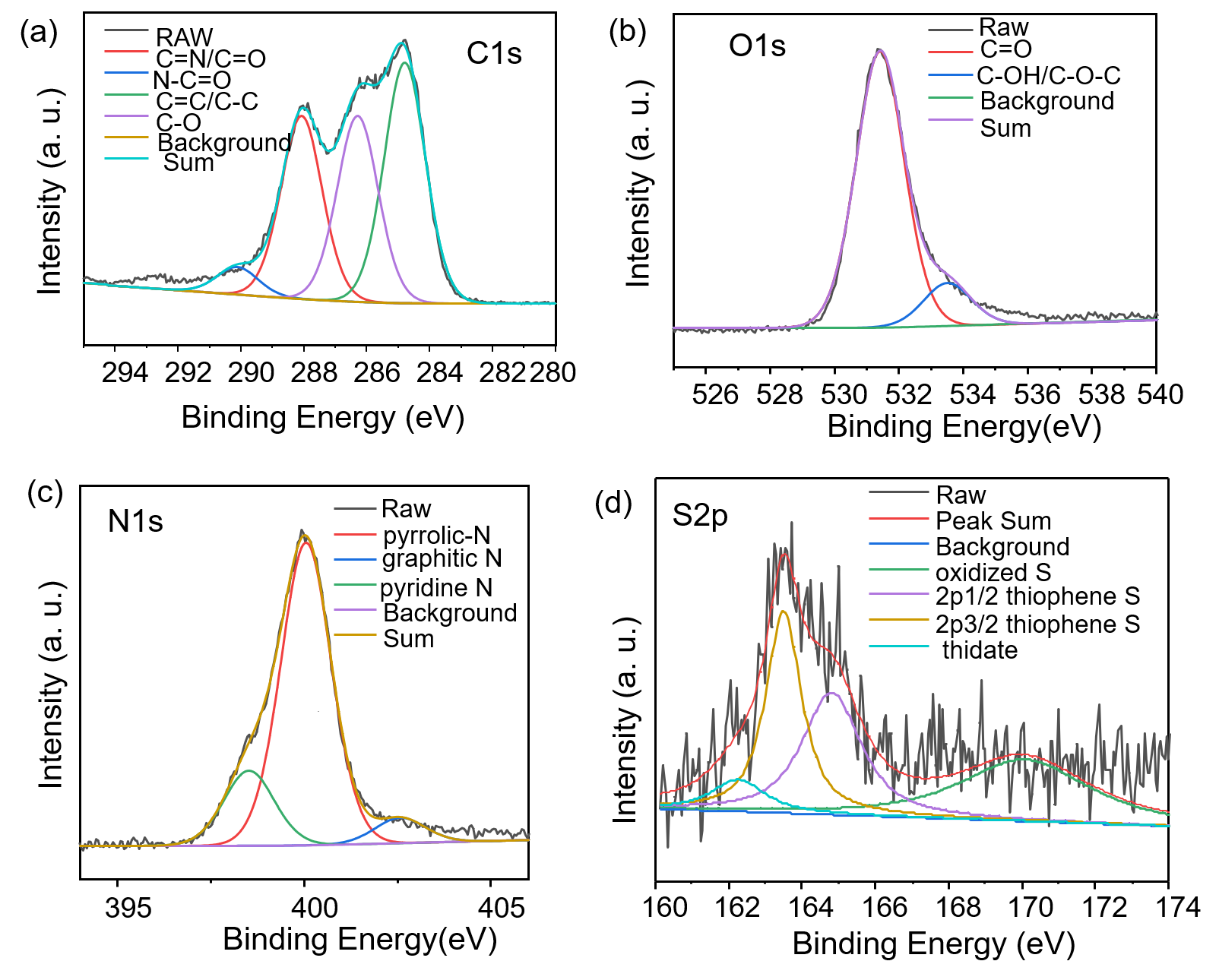

Supplement: Supplementary file 2 — Additional file 2: Fig. S2. High resolution XPS spectrum of C 1 s (a), N 1 s (b), O 1 s (c) and S 2p (d), respectively. [file 12951_2022_1271_MOESM2_ESM.png]

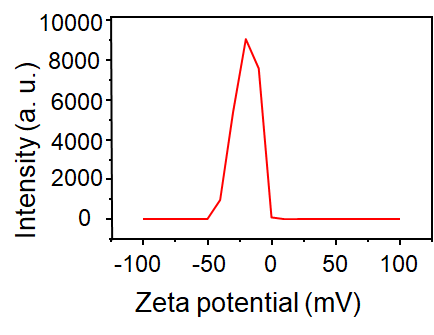

Supplement: Supplementary file 3 — Additional file 3: Fig. S3. Zeta potential measurement of the NIR-CDs. [file 12951_2022_1271_MOESM3_ESM.png]

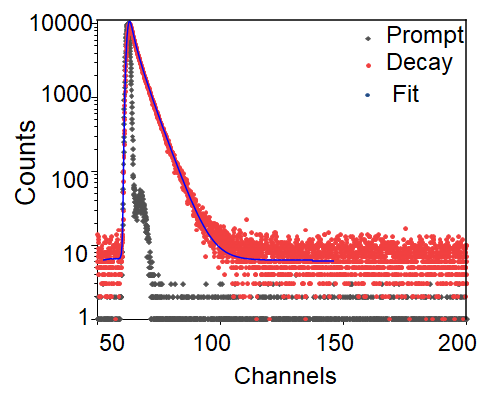

Supplement: Supplementary file 4 — Additional file 4: Fig. S4. Fluorescence delay curve of the NIR-CDs solution (20 µg/mL, λex: 450 nm, λem: 670 nm). [file 12951_2022_1271_MOESM4_ESM.png]

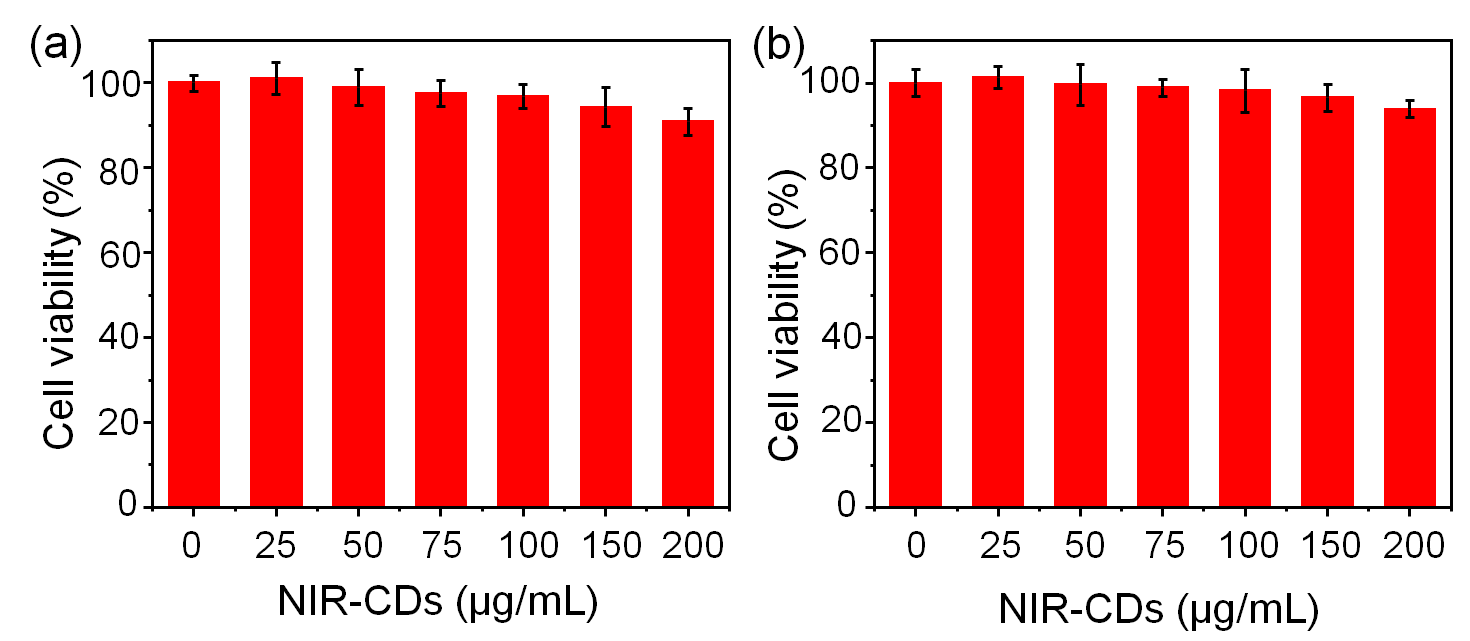

Supplement: Supplementary file 5 — Additional file 5: Fig. S5. Cytotoxicity assessment of the NIR-CDs via the standard MTT assay toward 16HBE and MCF-7 cells. [file 12951_2022_1271_MOESM5_ESM.png]

QC Sample

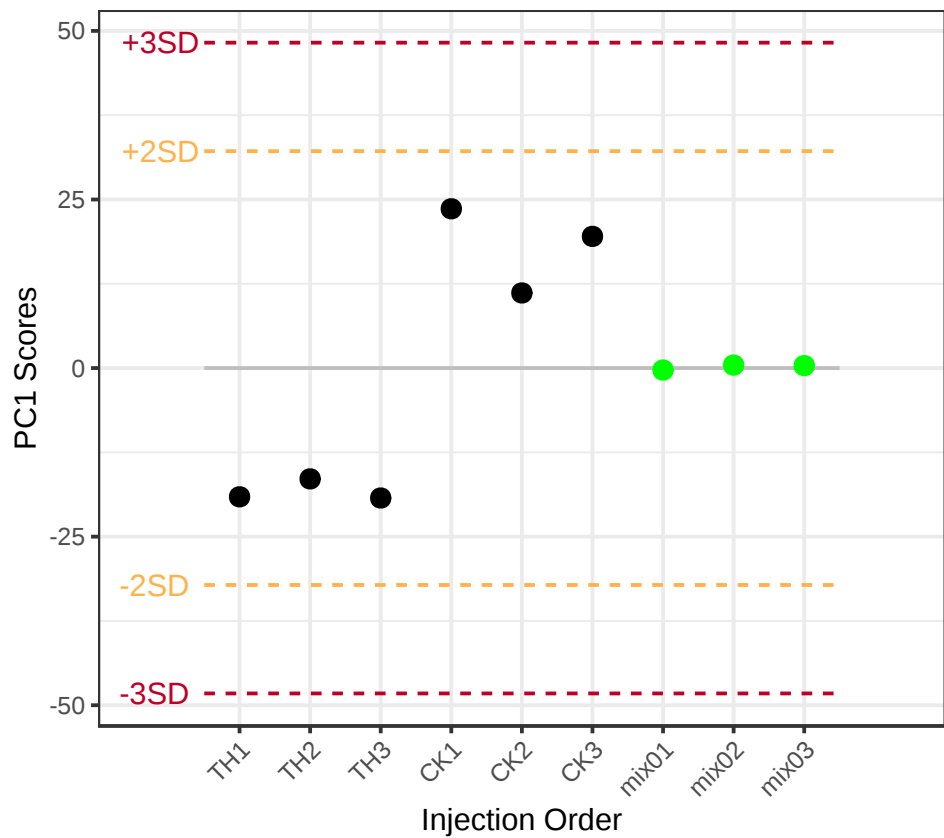

Supplement: Supplementary file 6 — Additional file 6: Fig. S6. Clustering relationship between gene modules. [file 12951_2022_1271_MOESM6_ESM.pdf]

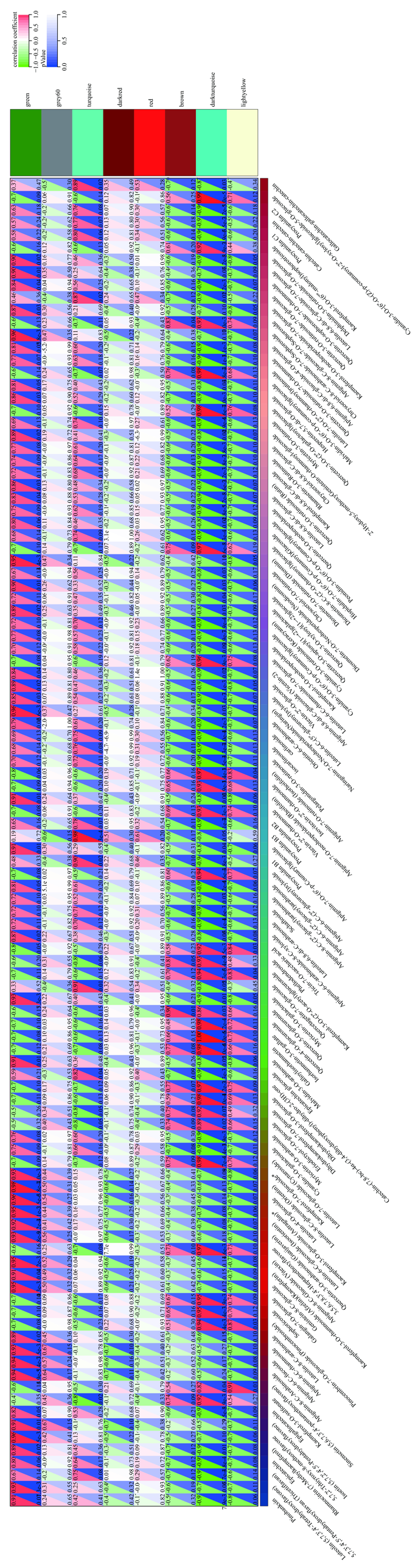

Supplement: Supplementary file 7 — Additional file 7: Fig. S7. Correlation heatmap between flavonoids content and module. [file 12951_2022_1271_MOESM7_ESM.jpg]

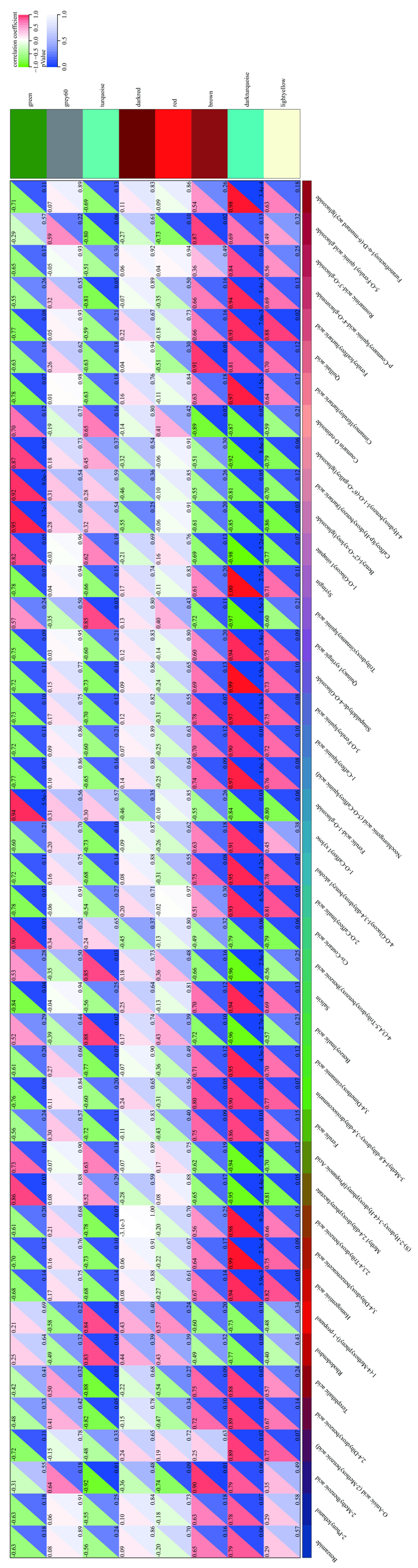

Supplement: Supplementary file 8 — Additional file 8: Fig. S8. Correlation heatmap between phenolic acids content and module. [file 12951_2022_1271_MOESM8_ESM.jpg]

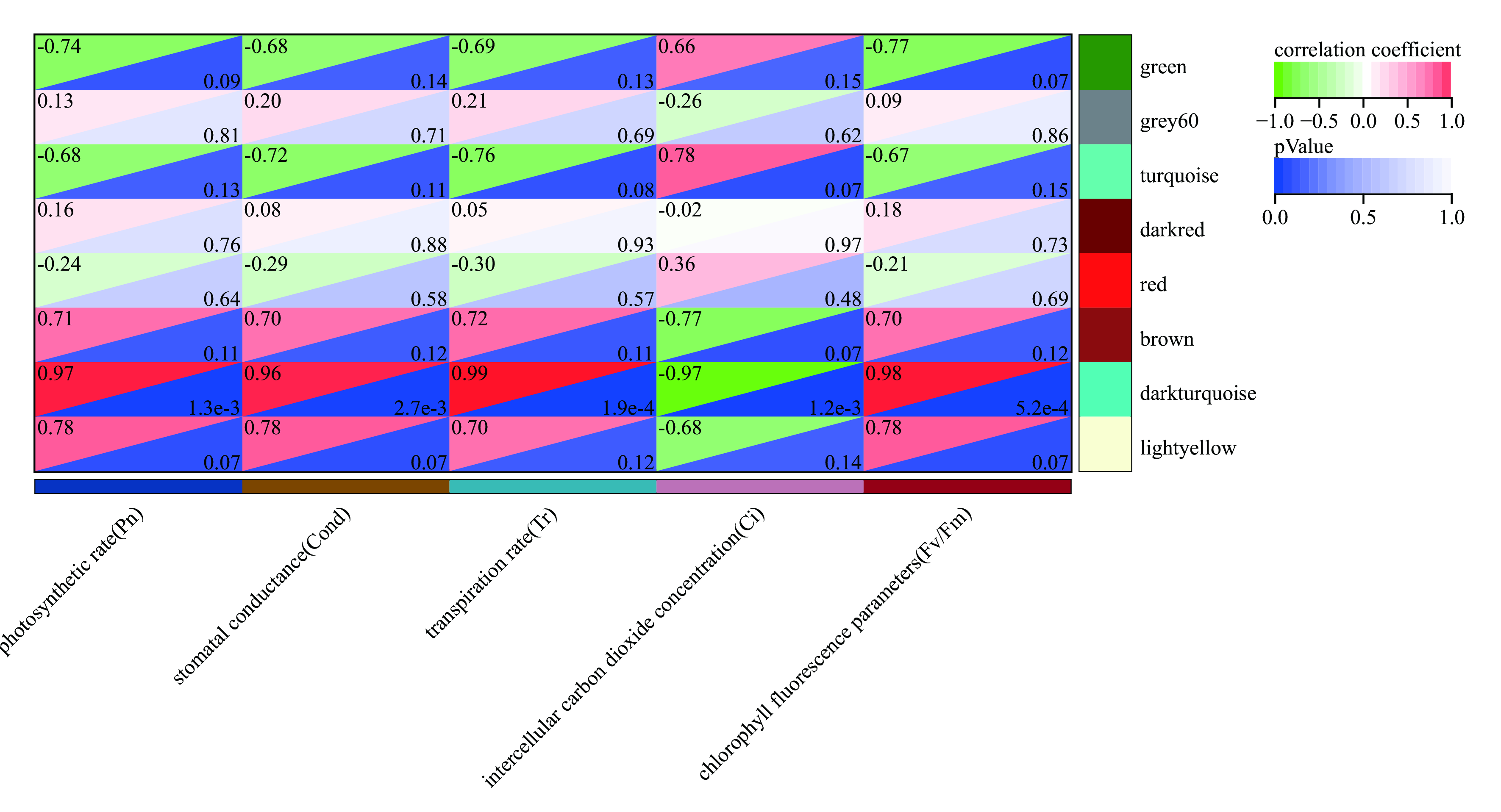

Supplement: Supplementary file 9 — Additional file 9: Fig. S9. Correlation heatmap between photosynthetic efficiency and module. [file 12951_2022_1271_MOESM9_ESM.tif]

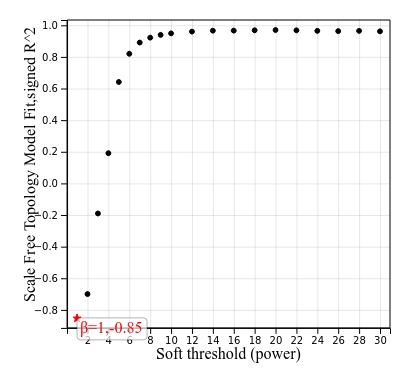

Supplement: Supplementary file 10 — Additional file 10: Fig. S10. Soft threshold figures for the weighted gene co-regulatory network analysis. [file 12951_2022_1271_MOESM10_ESM.zip › Additional files Fig. S10 A.jpeg]

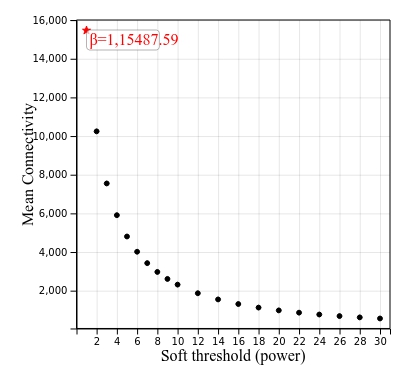

Supplement: Supplementary file 10 — Additional file 10: Fig. S10. Soft threshold figures for the weighted gene co-regulatory network analysis. [file 12951_2022_1271_MOESM10_ESM.zip › Additional files Fig. S10 B.jpeg]
